# Proteomic Responses of Switchgrass and Prairie Cordgrass to Senescence

**DOI:** 10.3389/fpls.2016.00293

**Published:** 2016-03-14

**Authors:** Bimal Paudel, Aayudh Das, Michaellong Tran, Arvid Boe, Nathan A. Palmer, Gautam Sarath, Jose L. Gonzalez-Hernandez, Paul J. Rushton, Jai S. Rohila

**Affiliations:** ^1^Department of Biology and Microbiology, South Dakota State UniversityBrookings, SD, USA; ^2^Department of Plant Science, South Dakota State UniversityBrookings, SD, USA; ^3^Grain, Forage and Bioenergy Research Unit, United States Department of Agriculture - Agricultural Research ServiceLincoln, NE, USA; ^4^22nd Century Group Inc.Clarence, NY, USA

**Keywords:** biofuel grasses, cordgrass, switchgrass, proteomics, senescence

## Abstract

Senescence in biofuel grasses is a critical issue because early senescence decreases potential biomass production by limiting aerial growth and development. 2-Dimensional, differential in-gel electrophoresis (2D-DIGE) followed by mass spectrometry of selected protein spots was used to evaluate differences between leaf proteomes of early (ES)- and late- senescing (LS) genotypes of Prairie cordgrass (ES/LS PCG) and switchgrass (ES/LS SG), just before and after senescence was initiated. Analysis of the manually filtered and statistically evaluated data indicated that 69 proteins were significantly differentially abundant across all comparisons, and a majority (41%) were associated with photosynthetic processes as determined by gene ontology analysis. Ten proteins were found in common between PCG and SG, and nine and 18 proteins were unique to PCG and SG respectively. Five of the 10 differentially abundant spots common to both species were increased in abundance, and five were decreased in abundance. Leaf proteomes of the LS genotypes of both grasses analyzed before senescence contained significantly higher abundances of a 14-3-3 like protein and a glutathione-S-transferase protein when compared to the ES genotypes, suggesting differential cellular metabolism in the LS vs. the ES genotypes. The higher abundance of 14-3-3 like proteins may be one factor that impacts the senescence process in both LS PCG and LS SG. Aconitase dehydratase was found in greater abundance in all four genotypes after the onset of senescence, consistent with literature reports from genetic and transcriptomic studies. A Rab protein of the Ras family of G proteins and an s-adenosylmethionine synthase were more abundant in ES PCG when compared with the LS PCG. In contrast, several proteins associated with photosynthesis and carbon assimilation were detected in greater abundance in LS PCG when compared to ES PCG, suggesting that a loss of these proteins potentially contributed to the ES phenotype in PCG. Overall, this study provides important data that can be utilized toward delaying senescence in both PCG and SG, and sets a foundational base for future improvement of perennial grass germplasm for greater aerial biomass productivity.

## Introduction

Prairie cordgrass (PCG) and switchgrass (SG) are two warm-season C_4_ grasses that are widely adapted to North American climatic conditions and have great potential as feedstock for the lignocellulosic-based biofuels industry (Lee and Boe, 2005; Sarath et al., 2008; Gonzalez-Hernandez et al., 2009). Senescence is a crucial plant development process that enables the remobilization of nutrients within a plant (Noodén et al., 1997). Leaf senescence is marked by the degradation of subcellular compartments, such as chloroplasts, and the remobilization of nutrients to other parts of the plants, such as seeds and underground rhizomes in the case of PCG and SG. The balance between oxidative stress and antioxidant activity plays a crucial role during senescence (Prochazkova et al., 2001). Senescence can be initiated by complex signals of age-specific factors in the genome and by temperature or day length in the case of seasonal senescence, and is often accompanied with increased ROS (reactive oxygen species) and oxidative stress (Jones et al., 2012; Palmer et al., 2015). Although ROS are generated continuously during normal growth, they are balanced by various antioxidant pathways, thus maintaining an optimal cellular redox state for growth. However, during stress related and age specific senescence, these antioxidant pathways cannot overcome oxidative stress, leading to senescence rather than normal growth. Mitochondria are among the major sources of ROS and ROS-related stress signals during programmed cell death (Zhao and Xu, 2000; Fleury et al., 2002).

The onset of early senescence, which is different from aging (Lim et al., 2003), is known to affect the amount of accumulated biomass by a perennial grass (Sarath et al., 2014). During senescence, leaves lose their photosynthetic capability sharply and, as a result, much less carbon is assimilated by the plant. Because leaves are the primary organ in plants that fixes carbon and directly contributes to plant biomass yields, untimely leaf senescence can cause losses in the total potential biomass yields of perennial grasses (Rinerson et al., 2015). Moreover, early senescing plants become more vulnerable to pathogen attacks, especially fungal pathogens, further reducing the biomass of the biofuel crops (Ahonsi et al., 2013). From an evolutionary point of view, senescence evolved to maximize the reutilization of nutrients that were accumulated by leaves during the growing season (Bleecker, 1998). Senescence in plants is a genetically programmed sequence of biochemical and physiological changes, but little is known about it at the protein level in bioenergy crops. Early and recent studies (Gan and Amasino, 1997; Biswal and Biswal, 1999; Palmer et al., 2014, 2015) suggested that senescence is highly correlated with the differential expression of senescence-associated-genes (SAGs). By controlling these signature genes, which regulate the juvenile to adult phase transition in plants, it may be possible to modify or enhance the biomass properties of a wide range of bioenergy feedstock (Chuck et al., 2011). Identifying and using new SAGs is therefore necessary in breeding programs for bioenergy feedstock crops.

A transcriptomic study of switchgrass flag leaf development from elongation through the onset of senescence was performed recently by Palmer et al. (2015). Many candidate genes were identified that were presumably involved in regulating the expression of senescence-related pathways. The authors found that during the onset of senescence, leaf chlorophyll content decline was associated with a significant upregulation in transcripts coding for enzymes involved in chlorophyll degradation and a large number of SAGs. Moreover, genes such as ureide, ammonium, nitrate, and molybdenum transporters that code for nitrogen and mineral utilization shared expression profiles that were significantly co-regulated with the expression profiles of NAC transcription factors. Similarly, Rinerson et al. (2015) identified 240 *WRKY* genes in the switchgrass genome and studied their expression during flag leaf development. Twenty-eight of these *WRKY* genes were identified as possible targets for increasing biomass yields in SG by delaying senescence. During senescence, protein activation by post-translational modification may be an additional mechanism of regulation. Several studies have shown that mRNA abundance for some genes may not necessarily be a predictor of protein abundance (Wang et al., 2006; Carp and Gepstein, 2007).

Similar to transcriptomics, high-throughput proteomic studies are a powerful tool to analyze changes in protein accumulation levels and post-translational modifications (Liu et al., 2013; Robbins et al., 2013; Wang et al., 2014), but no detailed proteomic study has been conducted on leaf senescence in either switchgrass or cordgrass.

Here, the power of global proteomics was utilized with an aim to profile and identify SG and PCG proteins that were associated with the senescence process using two contrasting genotypes for each species. Differentially abundant proteins in leaves from an early-senescence (ES) genotype were compared with those in a late-senescence (LS) genotype before and after the onset of senescence. Our results provide insights into the molecular basis of the differential responses of the two economically important bioenergy feedstock crops. These results may be instrumental in the rational engineering of senescence in SG and PCG with longer growing periods for increased biomass production.

## Materials and methods

### Plant materials and treatments

Leaf tissues were collected in triplicates from field-established clones of two different genotypes of SG [Genotype # 5 (ES SG), Genotype # 4 (LS SG). There was a 10-day difference between the two genotypes for date of onset of anthesis (10 August for the ES SG genotype compared 20 August for the LS SG genotype on average at Brookings, SD). These plants were selected from clonally maintained field nurseries established from random seedlings obtained from cultivar Sunburst; similarly, phenotypic selection from a larger PCG population was used to identify the ES PCG and LS PCG genotypes (Boe, unpublished). Similar to the phenological difference observed between the two switchgrass genotypes, there was about a 10-day difference in the onset of anthesis between the two PCG genotypes at the Brookings SD location. Leaves were collected from three clonal replicates of all four genotypes at two different times, before senescence and after senescence, for a total of 24 samples. Samples were assigned to groups A through H based on source population and harvest time (Table 1). The timing of senescence, for the sake of before and after senescence, was determined by measuring chlorophyll content (Palmer et al., 2015) using a hand-held chlorophyll meter (Supplementary Figure 1). After harvest, all leaf tissues were snap frozen under liquid nitrogen and were stored at −80°C until further use.

**Table 1:** **Plants, treatments, and groups for sample collection**.

**Genotype**	**Collection time**	**Group**	**Sample #**
Switchgrass clone # 5 (early senescence) ES SG	Before senescence	A	Sample #1
			Sample #2
			Sample #3
	After senescence	B	Sample #4
			Sample #5
			Sample #6
Switchgrass clone # 4 (late senescence) LS SG	Before senescence	C	Sample #7
			Sample #8
			Sample #9
	After senescence	D	Sample #10
			Sample #11
			Sample #12
Prairie cordgrass-ND (early senescence) ES PCG	Before senescence	E	Sample #13
			Sample #14
			Sample #15
	After senescence	F	Sample #16
			Sample #17
			Sample #18
Prairie cordgrass-SD (late senescence) LS PCG	Before senescence	G	Sample #19
			Sample #20
			Sample #21
	After senescence	H	Sample #22
			Sample #23
			Sample #24

Two genotypes of SG and two genotypes of PCG were selected with two treatments for each for sample collection. Samples were collected in triplicate.

### Sample preparation, 2-D differential in-gel electrophoresis (2D-DIGE), gel staining, image analysis, and protein identification by LC-MS/MS

The fold change in abundance of different proteins was analyzed in the form of ratios for eight different sets of comparisons based on group assignment (Table 1). Four are ratios of LS to ES PCG and SG: (i) the ratio of LS to ES PCG before senescence (G/E), (ii) the ratio of LS to ES PCG after senescence (H/F), (iii) the ratio for LS to ES SG before senescence (C/A), and (iv) the ratio of LS to ES SG after senescence (D/B). Similarly, the other four sets were as follows: (v) the ratio for after to before senescence in ES PCG (F/E), (vi) the ratio for after to before senescence in LS PCG (H/G), (vii) the ratio for after to before senescence in ES SG (B/A), and (viii) the ratio for after to before senescence in LS SG (D/C).

#### Protein lysate preparation

One gram of leaf tissue samples was ground to powder under liquid nitrogen using a mortar and pestle. Three hundred microliters of 2-D cell lysis buffer (30 mM Tris-HCl, pH 8.8, containing 7 M urea, 2 M thiourea, and 4% CHAPS) was added to this ground tissue and subjected to sonication on ice (Hurkman and Tanaka, 1986). Next, the tubes were shaken for 30 min on a shaker at room temperature. Then, samples were centrifuged for 30 min at 4°C at 25,000 × g, and the supernatant was collected. The concentration of protein was measured in the supernatant by Bio-Rad protein assay buffer with BSA as a standard following the standard manufacturer's guidelines (Peterson, 1983). Lysate samples were diluted with 2-D lysis buffer to a concentration of 5 mg/ml.

#### Minimal cy dye labeling

The 2D-DIGE were performed by Applied Biomics (Hayward, CA) following the protocol described in Robbins et al. (2013) and Das et al. (in press). Briefly, 1.0 μl of diluted Cy Dye was added to 30 μg of protein lysate (1:5 diluted with DMF from 1 nM/μl stock), followed by a short vortexing. The tubes were kept under dark on ice for 30 min followed by the addition of 1.0 μl of 10 mM lysine to each of the samples and vortexing; then, the reaction was kept in the dark on ice for additional 15 min. A pooled protein sample was prepared containing equal amounts of all 24 samples and was labeled with Cy2 using the same protocol. The Cy2 labeled sample was used as an internal control to compare gel-to-gel variations. For one gel, 3 samples consisting of Cy2, Cy3, and Cy5 labeled samples were mixed with 2X 2-D sample buffer (8 M urea, 4% CHAPS, 20 mg/ml DTT, 2% pharmalytes, and a trace amount of bromophenol blue). Then, 100 μl of destreak solution and rehydration buffer (7 M urea, 2 M thiourea, 4% CHAPS, 20 mg/ml DTT, 1% pharmalytes, and a trace amount of bromophenol blue) was also added to make a final volume of 350 μl for the 18 cm IPG strip (pH 3-10). This was mixed well and spun before loading the labeled samples into the strip holder.

#### IEF, SDS-PAGE, image scan and data analysis

After loading the labeled samples into the IPG strip holder, 18 cm strips were put facedown, and 1.5 ml of mineral oil was added on the top of the strips. This was followed by the protocol provided (Amersham BioSciences) and isoelectric focusing (IEF) was carried out in the dark at 20°C. After IEF, the IPG strips were incubated in freshly made equilibration buffer I (50 mM Tris-HCl, pH 8.8, containing 6 M urea, 30% glycerol, 2% SDS, a trace amount of bromophenol blue, and 10 mg/ml DTT) for 15 min with slow shaking. Then, the strips were rinsed in freshly prepared equilibration buffer II (50 mM Tris-HCl, pH 8.8, containing 6 M urea, 30% glycerol, 2% SDS, a trace amount of bromophenol blue, and 45 mg/ml iodacetamide) for 10 min with slow shaking. The strips were then rinsed in the SDS gel running buffer once, followed by their transfer into the SDS gel (12.5% acrylamide SDS gel prepared using low florescent glass plates). They were then sealed with 0.5% (w/v) agarose solution (in SDS gel running buffer). Running of the SDS gels was carried out at 15°C and stopped when the dye front ran out of the gels.

After the SDS-PAGE images were scanned using Typhoon TRIO (GE Healthcare Bioscience, Pittsburgh, PA, USA), and analysis of the scanned images was conducted using Image Quant software (version 6.0, GE Healthcare). An in-gel analysis of the images was conducted using DeCyder software, version 6.5 (GE Healthcare Bioscience). For this step, a difference in-gel analysis (DIA) tool made by DeCyder software was used. The value of the estimated number of spots was 3000. For the sake of low experimental variation, an automated tool made by DeCyder software was chosen for background subtraction and normalization of visible protein spots. The DIA datasets, along with the images, were put into the biological variation analysis (BVA) module made by DeCyder software. The spot intensity data were normalized with the internal standard sample that was labeled with Cy2 dye. The protein spots ratios that had increased or decreased abundance by 1.5-fold and had a Student's *t*-test value of *p* ≤ 0.05 were considered to be differentially abundant, along with the extra condition that the spot should be present and analyzed in all 2D gels. These ratios were calculated by the DeCyder image analysis software from spot volumes. As per the manufacturer's instructions the spot ratios were calculated as follows: (volume of secondary image spot/volume of primary image spot). This ratio indicated the change in spot volume between the two images. These ratio values were normalized, so that the modal peak of volume ratios was zero (since the majority of proteins are not up or down regulated). This ratio parameter is referred to as the volume ratio. In all DeCyder 2D Software DIA tables (Supplementary Tables 1, 2) the volume ratio is expressed in the range of 1–1,000,000 for increases in spot volumes and −1 to −1,000,000 for decreases in spot volumes. Values between −1 and 1 are not represented, hence a two-fold increase and decrease is represented by 2 and −2, respectively (and not 2 and 0.5 as might have been expected) (GE Healthcare Bio-Sciences, Pittsburgh, USA).

#### Spot picking and trypsin digestion and mass spectrometry

Protein spots that were statistically significant (see Section IEF, SDS-PAGE, Image Scan and Data Analysis) with a *p* ≤ 0.1 and cut off value of 1.5-fold were picked up using the Ettan Spot Picker (GE Healthcare). The picked gel spots were washed a few times, digested with modified porcine trypsin protease (Trypsin Gold, Promega), and then desalted using Zip-tip C18 (Millipore, Billerica, MA, USA) (Robbins et al., 2013; Gupta et al., 2014; Hayashi et al., 2015; Das et al., in press). Peptides were eluted from the Zip-tip with 0.5 μl of matrix solution (α-cyano-4-hydroxycinnamic acid, 5 mg/ml in 50% acetonitrile, 0.1% trifluoroacetic acid, and 25 mM ammonium bicarbonate) and spotted onto a MALDI plate (Robbins et al., 2013; Hayashi et al., 2015; Das et al., in press).

MALDI-TOF (MS) and TOF/TOF (tandem MS/MS) were performed on a 5800 mass spectrometer (AB Sciex). MALDI-TOF mass spectra were acquired in reflectron positive ion mode, averaging 2000 laser shots per spectrum. TOF/TOF tandem MS fragmentation spectra were acquired for each sample, averaging 2000 laser shots per fragmentation spectrum on each of the 10 most abundant ions present in each sample (excluding trypsin autolytic peptides and other known background ions) (Gupta et al., 2014; Hayashi et al., 2015; Das et al., in press).

#### Database search

Analysis of the MS/MS results were performed using GPS Explorer, version 3.5, which was equipped with a MASCOT search engine (Matrix science). A search in the database of the National Center for Biotechnology Information non-redundant (NCBInr) and in Phytozome v.10.3 (http://phytozome.jgi.doe.gov/pz/portal.html#!info?alias=Org_Pvirgatum) was performed without constraining the protein molecular weight or isoelectric point, with variable carbamidomethylation of cysteine, oxidation of methionine residues, and one missed cleavage allowed in the search parameters (Table 2 along with Supplementary Tables 5, 6). Candidates with either protein score C.I.% (Confidence Interval) or Ion C.I.% greater than 95 were considered significant.

**Table 2:** **Proteins identified by 2D-DIGE followed by Mass Spectrometry**.

**Spot number**	**Top ranked protein name**	**Accession No**.	**Protein MW (kDa)**	**Protein pI**
1	Phosphoenolpyruvate carboxylase 1	CAPP1_MAIZE	109.22	5.8
2	Putative aconitate hydratase	ACOC_ORYSJ	98.02	5.7
3	^**^Phosphoenolpyruvate carboxylase	gi|154146924	49.67	0
4	Polyphosphate kinase	gi|116513638	83.23	5.8
5	Uncharacterized protein	gi|293332375	90.40	5.7
6	^**^Phosphoenolpyruvate carboxylase	gi|11991545	108.44	0
11	^**^Phosphoenolpyruvate carboxykinase	gi|220938712	55.61	0
13	PREDICTED: fibulin-2	gi|125829574	92.63	5.4
14, 264	^*^Phosphoenolpyruvate carboxylase	gi|154146806	49.30	0
15, 16, 19	^*^Transketolase, chloroplastic	TKTC_MAIZE	72.94	5.5
23	Pyruvate orthophosphate dikinase	gi|380691814	74.79	0
24	^**^V-type proton ATPase catalytic subunit A	VATA_MAIZE	61.91	5.9
25	2,3-bisphosphoglycerate-independent phosphoglycerate mutase	PMGI_MAIZE	60.58	5.3
31	Succinate dehydrogenase [ubiquinone] flavoprotein subunit	DHSA_ORYSJ	68.81	6.6
33, 34, 35, 36	^*^Phosphoenolpyruvate carboxykinase	gi|220938712	55.61	7.6
38	ATP synthase subunit alpha	ATPA_SACOF	55.65	5.9
43	^**^Phosphoenolpyruvate carboxylase	gi|154146744	49.43	0.0
44	ATP synthase subunit alpha, mitochondrial	ATPAM_PHAVU	55.31	6.5
45, 46, 48	^*^ATP synthase subunit alpha, chloroplastic	ATPA_SACOF	55.65	5.9
49	ATP synthase subunit alpha, mitochondrial	ATPAM_ORYSI	55.33	5.9
53, 55	^*^ATP synthase subunit beta, chloroplastic	ATPB_SORBI	53.97	5.3
56, 127	^*^Ribulose bisphosphate carboxylase large chain	RBL_SECDI	51.60	6.2
58, 59, 70	^*^Ribulose bisphosphate carboxylase large chain	RBL_SETIT	52.64	6.4
60, 69, 286	^*^Ribulose bisphosphate carboxylase large chain	RBL_AVESA	52.90	5.9
65	^#^Probable sucrose-phosphate synthase 1	SPSA1_CRAPL	118.95	6.1
97, 120	^*^Cysteine protease 1 precursor	gi|226496089	50.73	5.0
98	Sedoheptulose-1,7-bisphosphatase, chloroplastic	S17P_WHEAT	42.03	6.0
115	Fructose-bisphosphate aldolase, chloroplastic	ALFC_ORYSJ	41.98	6.4
128	Unknown	gi|223974857	39.04	7.9
129	^**^Chain A, Leaf Ferredoxin-Nadp+ Reductase I	gi|427930675	34.80	0
133	Oxygen-evolving enhancer protein 1, chloroplastic	PSBO_HELAN	34.20	5.4
134	Oxygen-evolving enhancer protein 1, chloroplastic	PSBO_SOLLC	34.92	5.9
140	^**^Glutathione S-transferase GSTF14	gi|46276327	30.71	0
142	^**^NAD-dependent epimerase/dehydratase	gi|226499246	27.75	0
144	^**^PREDICTED-proteasome subunit alpha type-7-B isoform 1	gi|357159679	27.28	0
146	^**^Predicted protein	gi|255082634	69.94	0
147	Beta-ketoadipyl CoA thiolase	gi|171057883	41.78	7.6
208	Hypothetical protein azo3784	gi|119900073	12.28	8.2
209	Ribulose bisphosphate carboxylase large chain	RBL_APHSI	51.55	6.0
221, 279	^*^^$^Putative cytochrome c oxidase subunit II PS17	PS17_PINST	1.70	9.6
225	Oxysterol-binding protein-related protein 1D	ORP1D_ARATH	92.27	6.1
226	cytochrome b6-f complex iron-sulfur subunit	gi|195612712	23.97	8.5
230	S-adenosylmethionine synthase	METK1_BRAJU	43.19	5.5
237	Protein CYPRO4	CYPR4_CYNCA	55.61	7.6
273, 278	^*^Ras-related protein RABB1b	RAB1B_ARATH	23.16	6.5
275	14-3-3-like protein	1433_PEA	29.31	4.7
284	Ribulose bisphosphate carboxylase large chain	RBL_LIQST	52.62	6.0
287	^**^Predicted protein	gi|326529055	44.22	0
291	Cysteine proteinase Mir3 precursor	gi|162463464	51.75	5.8
298	^**^Receptor-like protein kinase	gi|2832304	46.55	0
301	^**^Auxin-binding protein ABP19a-like precursor	gi|359806878	22.23	0
311	^**^Hypothetical protein ZEAMMB73_006777	gi|413933720	6.12	0
313	^**^P1B-ATPase heavy-metal transporter	gi|315623028	115.92	0
314	^**^Chloroplast PSI type III chlorophyll a/b-binding protein	gi|159138869	13.85	0
316	^**^Hypothetical protein VITISV_010874	gi|147843505	37.03	0
317	^**^Glutathione S-transferase F8	gi|357114170	25.81	0
321	^**^Polyamine oxidase	gi|14485487	56.47	0

* *Different spot number corresponds to the same protein*.

** *pI value 0 corresponds to the proteins with isoelectric point either below 3.0 or above 10.0 or it's mixed up with a nearby spot, thus pI value is unspecific*.

# Largest protein based on molecular weight;

$ *Smallest protein based on MW*.

### Bioinformatic analyses

The best hit proteins from MS/MS were blasted to the NCBI (http://www.ncbi.nlm.nih.gov/) and Phytozome (Goodstein et al., 2012) databases. NCBI, Arabidopsis Information Resource (http://arabidopsis.org) (Rhee et al., 2003), and Uniprot (http://uniprot.org) (The UniProt Consortium, 2008) were used to retrieve further information on protein functions. The Kyoto Encyclopedia of Genes and Genomes website (KEGG; http://www.genome.jp/kegg/) (Kanehisa and Goto, 2000) was used to retrieve information on proteins regarding their involvement in metabolic pathways. STRING database, version 9.1 (http://string-db.org/) (Szklarczyk et al., 2011), was used to predict protein-protein interactions. We retrieved primary interaction data from the STRING database and portrayed the interaction in Cytoscape 3.1.1 software, along with a categorization of their functions (Shannon et al., 2003).

## Results and discussion

### Genotypes of SG and PCG with contrasting senescence phenotypes

The two contrasting genotypes of switchgrass selected from “Sunburst” differed in morphological and phenological traits. The LS genotype headed approximately 10 days later than the ES genotype and reached full senescence 2–3 weeks later in autumn. The LS genotype was more disease resistant, taller, and produced more average biomass yields (12.2 Mg dry matter ha^−1^ compared with 8.5 Mg dry matter ha^−1^) than the ES genotype.

Similar differences were found between the LS PCG (origin in southeastern South Dakota) and the ES PCG (origin in southeastern North Dakota). The LS PCG headed approximately 2 weeks later and reached full senescence approximately 2–3 weeks later in autumn than the populations PCG plants. The LS PCG genotype was taller (2.5 m) than the ES PCG plants (1.25 m) at its maximum height during anthesis. At peak standing in mid-summer, ES PCG plants of PCG produced an average of 7.1 Mg dry matter ha^−1^ compared with 15.4 Mg dry matter ha^−1^ for the LS PCG plants.

### Proteomic response during pre- and post-senescence in PCG and SG

**Figure 1:**
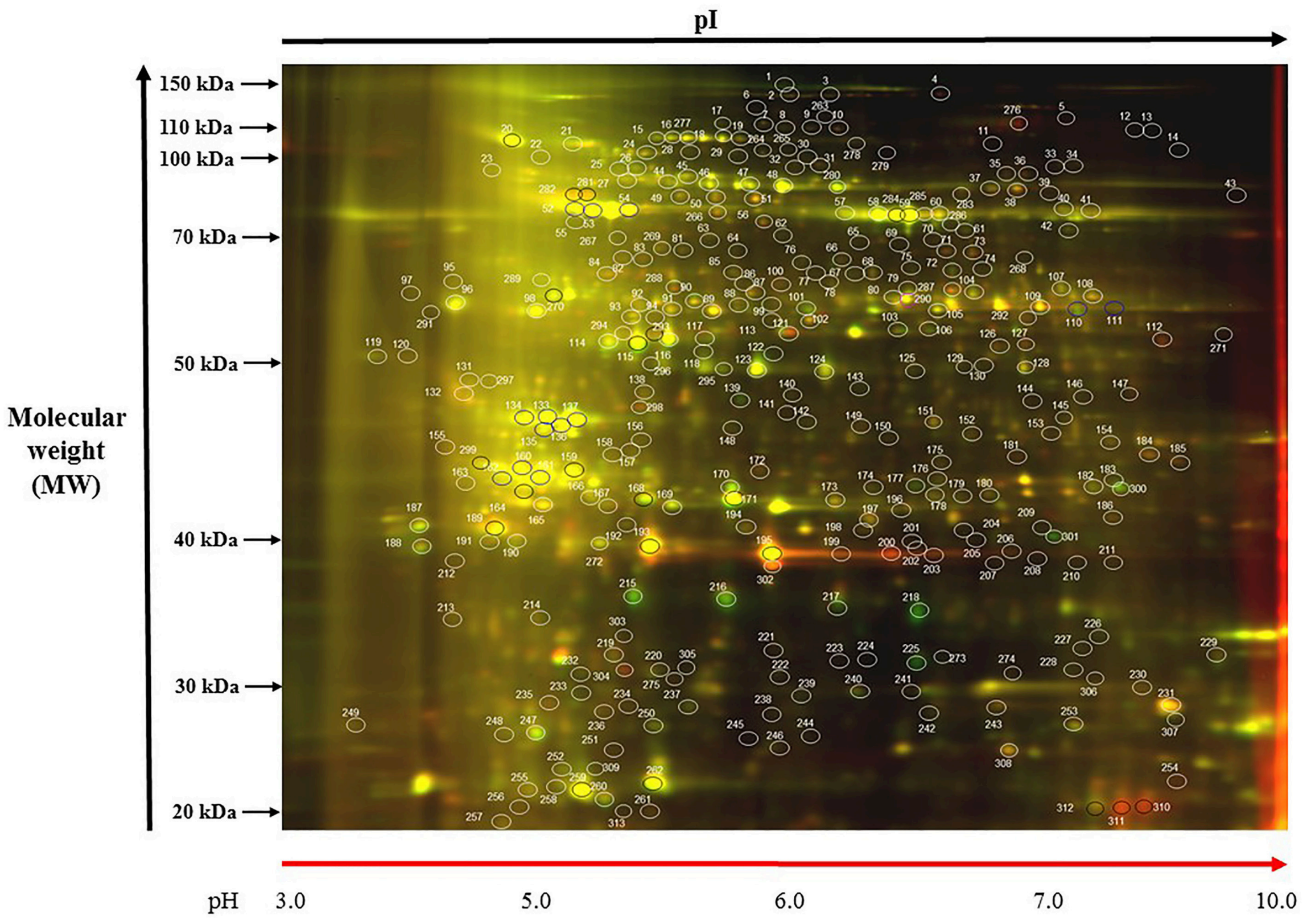
**Representative gel image: (A1/C7)**. Protein samples of SG and PCG leaves, control and treatment samples, were labeled with Cy3 (green) and Cy5 (red), respectively, and mixed in equal ratios. The first-dimension isoelectric points (pI, ranges from 3 to 10) and second-dimension molecular mass (in kDa and ranges from 20 to 150 kDa) are noted. Color coding: green spots indicate protein abundance is high in Cy3, red spots indicate protein abundance is high in Cy5, yellow spots indicates where protein abundance is similar in both the cases.

**Figure 2:**
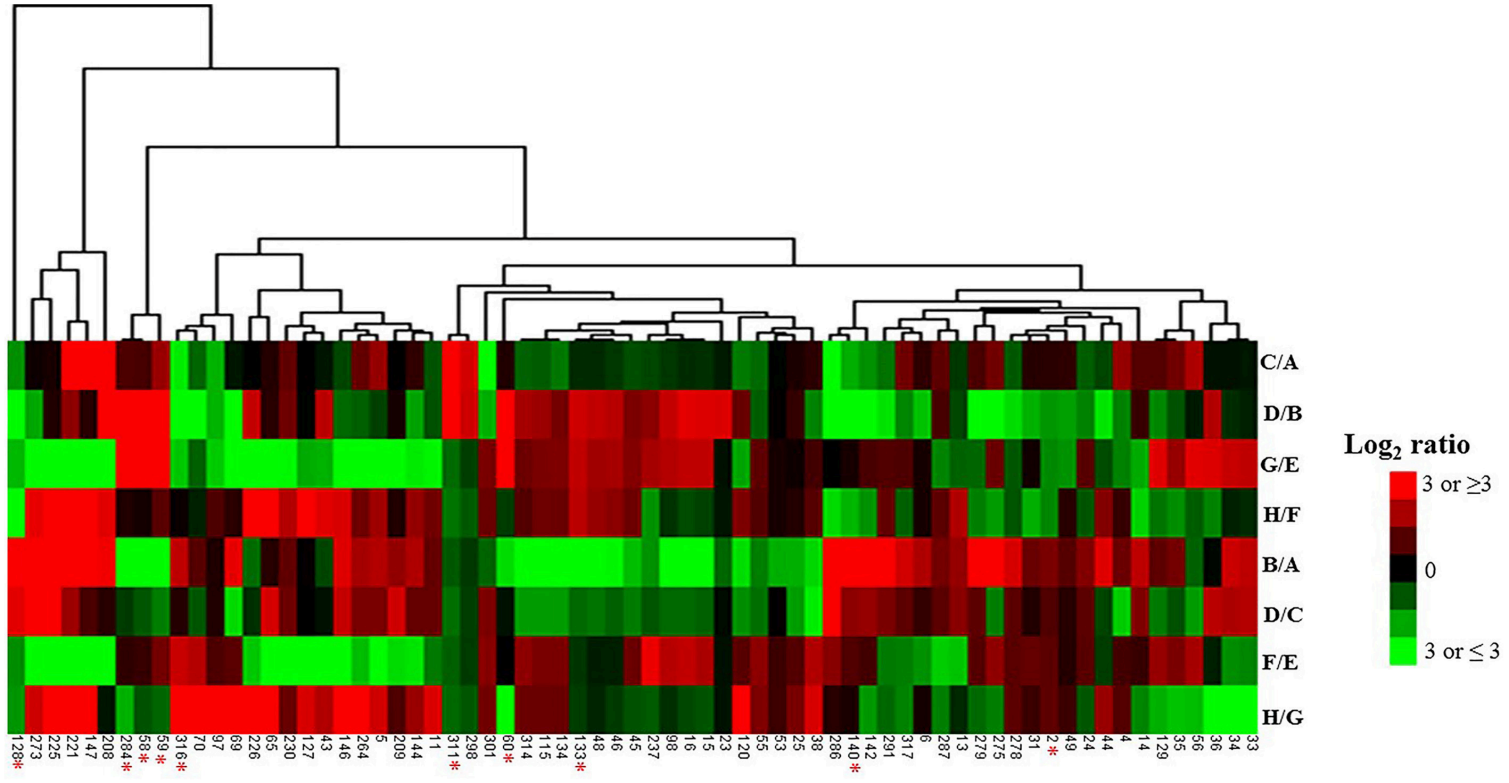
**Heat map of 69 protein identified as differentially abundant during senescence**. Heat map of proteins associated with senescence in eight different experiment groups. C/A, Before senescence (late/early SG); D/B, After senescence (late/early SG); G/E, Before senescence (late/early PCG); H/F, After senescence (late/early PCG); B/A, Early SG (after/before senescence); D/C, Late SG (after/before senescence); F/E, Early PCG (after/before senescence); H/G, Late PCG (after/before senescence). Hierarchical clustering was done as described in the method Section Bioinformatic Analyses. ^*^Means selected 10 protein spots (7 different proteins) showing common abundance pattern between SG and PCG when analyzing the fold change of protein abundance for after to before senescence.

A representative 2D-DIGE gel image of sample A1/C7 is shown in Figure 1. 2D-DIGE along with MS/MS revealed the differential abundance of 74 protein spots (Figure 1). Among those, 69 different protein spots were selected based on statistical analyses (threshold of significance of *p* ≤ 0.1) and a cut off value of 1.5-fold increase/decrease in protein abundance, which is shown in the heat map (Figure 2).

**Figure 3:**
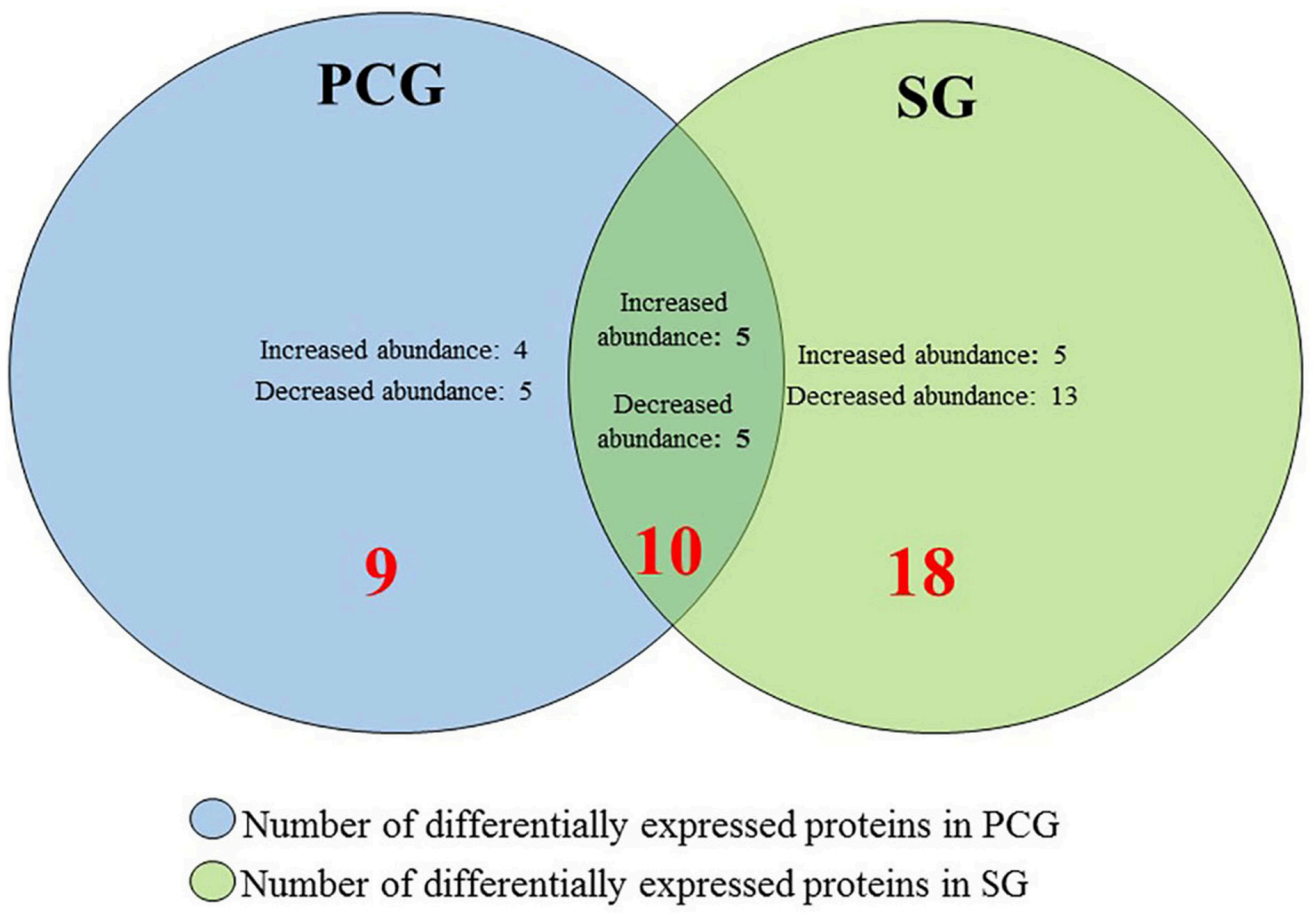
**Number of differentially abundant proteins in PCG and SG during senescence when the fold change of proteins was observed after/before senescence**. 19 proteins are differentially abundant in PCG, whereas 28 proteins are differentially abundant in SG. Among those proteins 10 proteins are common in both PCG and SG.

To achieve an improved understanding of the proteomic responses due to senescence, differentially abundant proteins that were identified in both PCG and SG were categorized according to their GO annotation function (Figure 3; Camon et al., 2003). Our analysis indicated that 69 differentially abundant proteins were found to be involved in various biological processes, including photosynthesis (41%), amino acid metabolism (13%), carbohydrate metabolism (12%), ATP synthesis (6%), protein metabolism (5%), kinase activity (4%), ATP signal transduction (3%), cell division (3%), sterol metabolism (1%), Auxin binding (1%), and GTPase signal transduction (1%).

**Figure 4:**
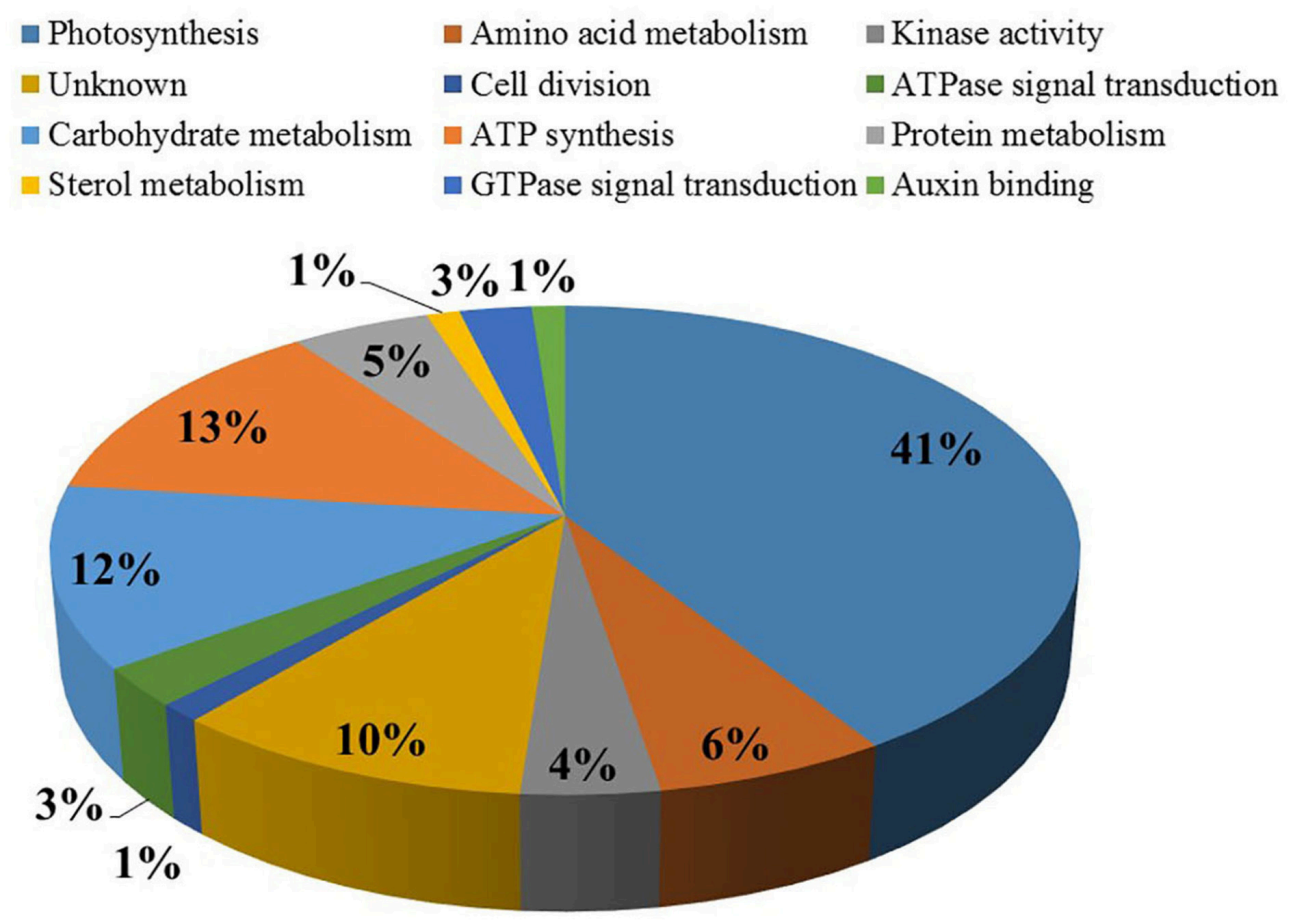
**Differentially abundant proteins, from the eight different comparisons in Figure 2, were categorized based on their Gene Ontology annotations**. The most abundant ontologies are shown. Percentage value symbolize abundance of proteins in each functional class.

When we analyzed the fold change in protein abundance for after-to-before senescence in LS and ES PCG, nine unique protein spots were found to be differentially abundant that were absent in SG (Supplementary Table 1), whereas ten protein spots were found to have common abundance patterns between SG and PCG (Figure 4). Among those 19 protein spots of interest, 9 had higher abundance, and 10 had lower abundance. Our proteomic profiling of both ES and LS SG genotypes revealed that 28 major protein spots were differentially abundant (Supplementary Table 2). Among these 28 protein spots, 18 proteins were found exclusively in SG, whereas the other 10 protein spots were found in both SG and PCG (Figure 3). Overall, 10 protein spots had increased and 18 protein spots had decreased abundance in SG.

To investigate the changes in the protein profiles of before—and after the onset of senescence (four different comparisons: B/A, D/C, F/E, and H/G) in PCG and SG (Table 1), we identified 10 major differentially abundant protein spots based on statistical significance (*p* ≤ 0.05) (seven different proteins: putative aconitate hydratase, ribulose bisphosphate carboxylase large chain, oxygen-evolving enhancer protein 1, glutathione S-transferase, hypothetical protein ZEAMMB73, and hypothetical protein VITISV) (Figure 2). Five protein spots were found to have higher abundance and five spots had lower abundance, most likely in response to senescence processes.

### Biological implications of selected proteins

#### Role of putative aconitate hydratase during senescence

**Figure 5:**
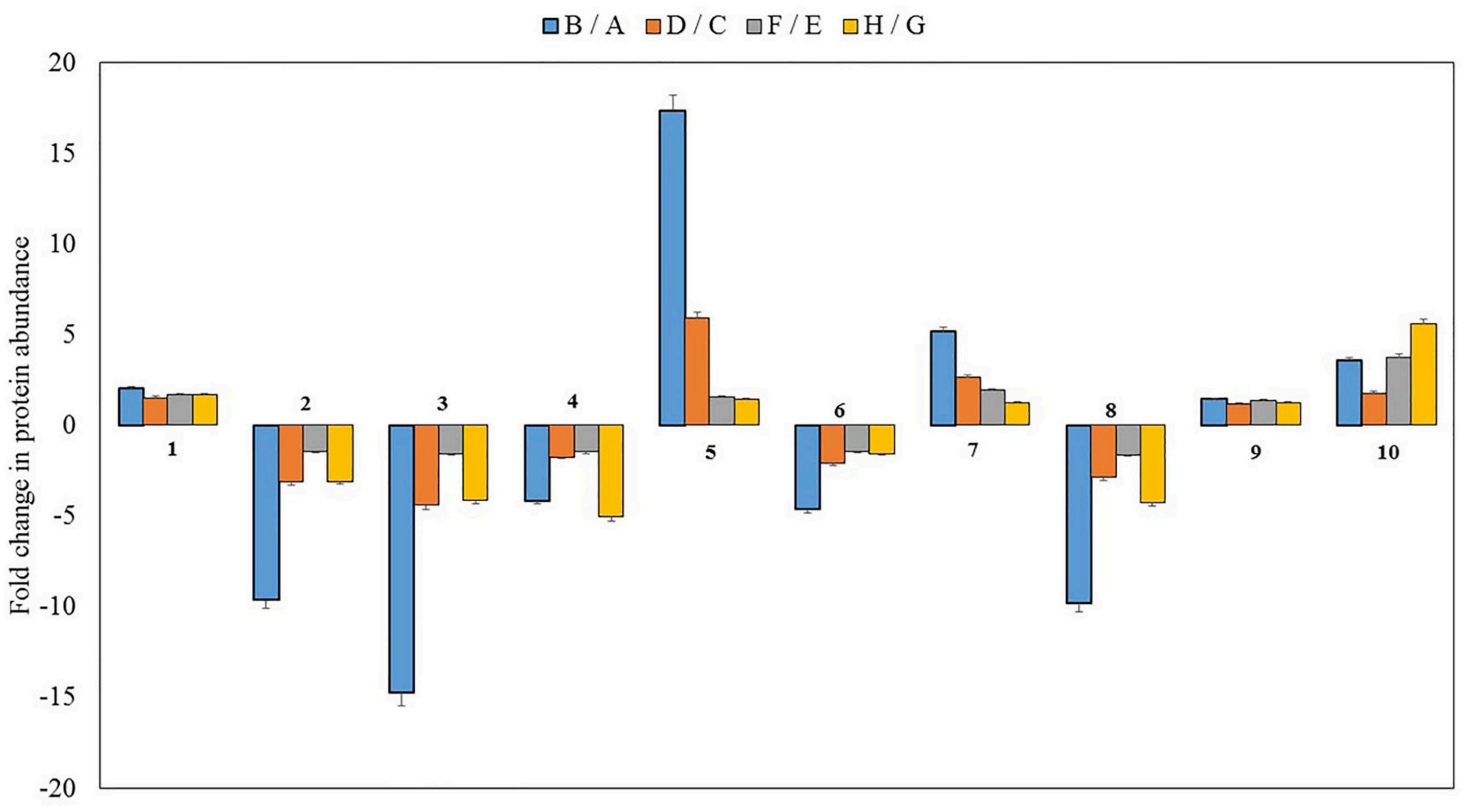
**Fold change of 10 differentially abundant proteins in the after to before senescence comparisons among the four different genotypes (*p* < 0.01)**. B/A, Early SG (after/before senescence; D/C, Late SG (after/before senescence); F/E, Early PCG (after/before senescence); H/G, Late PCG (after/before senescence). 1- putative aconitate hydratase, 2, 3, 4, 8- ribulose bisphosphate carboxylase large chain, 5- unknown protein of Zea mays, 6- oxygen-evolving enhancer protein 1, 7- a glutathione S-transferase, 9- hypothetical protein, 10- hypothetical protein VITISV. Standard error was calculated and displayed with error bar.

Putative aconitate hydratase was found to have increased abundance during senescence in all four comparisons (Figure 5). Aconitate hydratase catalyzes steps in the TCA cycle and glyoxylate cycle that isomerizes citrate to isocitrate (Evans et al., 1966). Sugar metabolism toward gluconeogenesis, hexose formation, and conversion to sucrose seems to be an important phenomenon to signal the source-sink translocation in perennial grasses (Supplementary Figure 2). Aconitate hydratase in *Arabidopsis* is reported to also bind mRNA of CSD2 (CuZn superoxide dismutase 2), which was found to play a key role in antioxidant defense mechanisms (Gregersen and Holm, 2007; Moeder et al., 2007). Moreover, reduced aconitate hydratase levels in cells reportedly enhanced resistance to oxidative stress. It was also reported that upregulation of the cytoplasmic aconitate hydratase gene in wheat flag leaf plays a crucial role in the mitochondria with respect to oxidative cell damage (Gregersen and Holm, 2007). Therefore, it is possible that aconitate hydratase in both SG and PCG helps to mediate oxidative stress and regulate plant growth or senescence by influencing the expression of genes for redox balance (Moeder et al., 2007). Two genes encoding aconitate hydratases were significantly upregulated in senescing SG flag leaves (Palmer et al., 2015), corroborating the findings at the protein level.

#### Role of glutathione s transferase in anti-oxidation and its early senescing and late senescing response in prairie cordgrass

It has been reported that GST is a marker gene for oxidative stress and that it participates in cellular protection when plants are subjected to wounding, pathogen attack, and lipid peroxidation (Bilang and Sturm, 1995; Grant et al., 2000). Consistent with previous findings, our proteomic analysis also showed significantly higher levels (five-fold) of a GST protein when its fold change was analyzed before and after senescence in the ES SG genotype (Figure 5).

**Figure 6:**
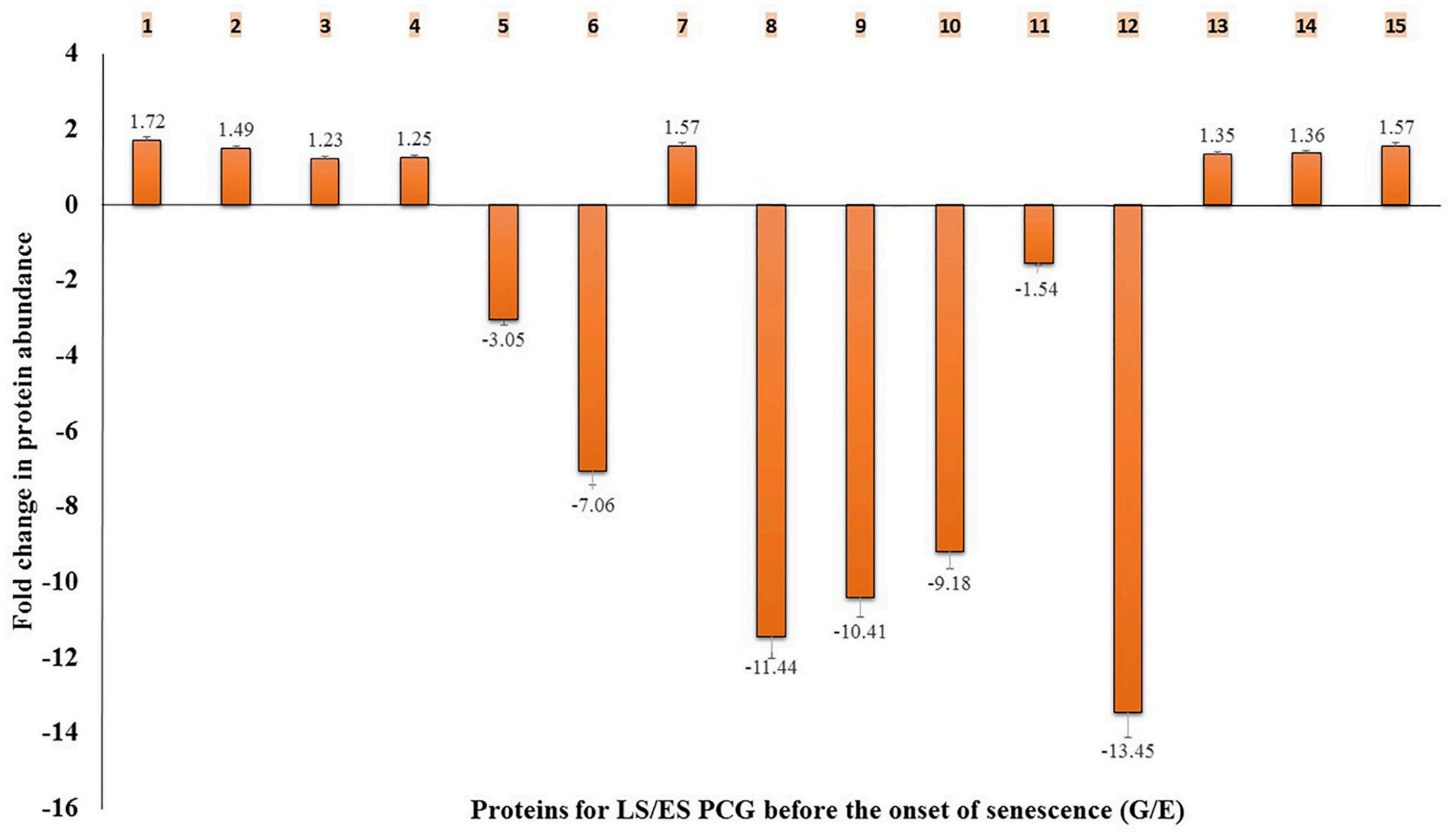
**Fold change of abundance in 15 different proteins for LS/ES PCG before the onset of senescence (G/E)**. 1- transketolase, 2- sedoheptulose-1,7-bisphosphatase, 3- fructose-bisphosphate aldolase, 4- glutathione S-transferase GSTF14, 5- S-adenosylmethionine synthase, 6- Ras-related protein, 7-14-3-3-like protein, 8- oxysterol-binding protein-related protein, 9- probable sucrose-phosphate synthase, 10- beta-ketoadipyl CoA thiolase, 11- cysteine protease 1 precursor, 12- putative cytochrome c oxidase subunit II PS17, 13- ATP synthase subunit alpha, 14- ATP synthase subunit beta, 15- oxygen-evolving enhancer protein 1. Standard error was calculated and displayed with error bar.

Our results also showed that a GST was found to be significantly higher in LS PCG before senescence than ES PCG (Figure 6). However, after senescence, the levels were higher in ES PCG than LS PCG (Supplementary Table 1), similar to what was observed for SG. This observation suggests that consistent higher abundance of GST levels in PCG before the onset of senescence may play a key role in delaying senescence signals by potentially delaying ROS mediated signaling. On the other hand, we found increased levels of this GST during senescence when we compared GST levels from after-to-before senescence in four different sets of comparisons (B/A, D/C, F/E, and H/G). These results imply that the levels of this specific GST increased during senescence. ROS are continuously generated in plants in different organelles, such as mitochondria, chloroplasts, lysosomes, and peroxisomes, but are eliminated by different mechanisms to keep a balance between generation and removal. GST plays a crucial role in the removal of reactive oxygen species. Major pathways involving GST retrieved from KEGG indicate that GST seems to be a crucial enzyme in balancing cellular oxidative stress.

#### Proteins involved in the regulation of photosynthesis during pre- and post-senescence

It is known that photosynthetic activity declines rapidly during leaf senescence (Kura-Hotta et al., 1987). Previous studies in rice showed that RuBisCO is highly active during leaf expansion but drastically reduced during senescence (Suzuki et al., 2001). Based on our 2-D DIGE analysis in SG and PCG, we also found significantly reduced levels of RuBisCO large subunit when the fold changes for this protein were compared before and after senescence in both PCG and SG genotypes (Figure 5). RuBisCO is the major enzyme responsible for assimilating carbon dioxide (Spreitzer and Salvucci, 2002). Reduced RuBisCO content can be expected to reduce CO_2_ fixation and net photosynthesis. It has been reported that RuBisCO is the prime target for degradation as a consequence of increased levels of ROS in chloroplasts (Khanna-Chopra, 2012).

Furthermore, 2D-DIGE analysis in the current investigation revealed that other proteins involved in photosynthesis, such as oxygen evolving enhancer (OEC) protein in photosystem II, ferrodoxin-NADP-reductase, and the α- and β-subunits of ATP synthase, had higher abundance in the LS PCG compared with the ES PCG genotype, critically when protein fold changes were compared before senescence. All of these proteins are involved in electron transfers or in ATP synthesis in chloroplasts, (Haehnel, 1984). Consistent higher abundance of these proteins in the LS PCG compared with the ES PCG before senescence suggests that an overall upregulated machinery of photosynthesis in plants may contribute to delaying the senescence process (Supplementary Figure 3). In line with our findings in PCG, downregulation of OEC has also been found to decrease photosynthesis in mutant *Arabidopsis* plants where one of the *psbO* genes was defective (Hager et al., 2002; Ifuku et al., 2005).

Higher abundance of the enzymes involved in photosynthetic electron transfer and ATP synthesis, along with the increased abundance of enzymes involved in the Calvin cycle, seems to contribute to the accumulation of higher biomass in LS PCG compared with ES PCG. However, consistent increased abundance of the photosynthetic machinery itself may have contributed to delay senescence in the LS genotypes. Photosystem II and its corresponding OEC proteins are tightly regulated during senescence (Lim et al., 2007). Downregulation of *OEC* gene has been known to reduce photosynthetic activity in mutant *Arabidopsis* plants, indicating that OEC protein plays a vital role in photosynthesis by oxidizing water in photosystem II on thylakoid membranes (Lundin et al., 2007; Dwyer et al., 2012). Interestingly, our proteomic evaluation showed that the levels of OEC proteins were reduced after the onset senescence compared with before senescence in PCG leaves and similar results were observed in SG as well (Figure 5). Although why *OEC* is downregulated during senescence remains unknown, it is clear that downregulation of *OEC* leads to photo-damage in photosystem II, with the malfunctioning of acceptor and donor electron carriers (Allahverdiyeva et al., 2009). This photo-damage may play a role in producing more ROS, thus destroying chlorophylls and RuBisCO large subunit and signaling the initiation of senescence. Interestingly, higher abundance of ferrodoxin NADP-reductase and the chloroplastic α- and β-subunits of ATP synthase in the LS PCG compared with the ES PCG also supports the idea that increased abundance of photosynthesis-related proteins may contribute to delay the senescence process in this plant.

#### Role of 14-3-3 like protein, s-adenosylmethionine synthase, and ras-related RAB protein in senescence signaling

The 14-3-3 like proteins are well studied and are involved in apoptotic and cell survival signaling in *Arabidopsis* (van Hemert et al., 2001). Similarly, S-adenosylmethionine (SAM) synthase generates SAM which is involved in the ethylene synthesis pathway and in many methylation-dependent pathways. The involvement of RAB protein in vesicular transport mechanisms of cells is well documented (Grbić and Bleecker, 1995; Fu et al., 2000; Woollard and Moore, 2008). We found differential abundance of these proteins in LS PCG to ES PCG (G/E) before senescence (Figure 6).

We observed that a 14-3-3 like protein was highly abundant in LS PCG compared with ES PCG before senescence, whereas SAM synthase and RAB protein had lower abundance. Earlier studies showed that a 14-3-3 protein antagonizes pro-apoptotic activity by playing a key role in integrating the signals for cell death and survival (Fu et al., 2000). It has been reported that the overexpression of the Arabidopsis 14-3-3 gene *GF14*λ in cotton plants resulted in maintaining green plants and significantly delaying senescence and improving drought stress tolerance (Yan et al., 2004; Gregersen et al., 2013). Similarly, the overexpression of a 14-3-3 gene in potato plants delayed senescence and elevated antioxidant activity, whereas the downregulation of this gene led to early senescence (Wilczyñski et al., 1998; Łukaszewicz et al., 2002). Thus, the overexpression of genes encoding 14-3-3 proteins with appropriate promoters in PCG may be a good strategy to delay senescence. Studies on RAB genes related to senescence are very limited, but their overexpression is known to accelerate leaf senescence in mutant Arabidopsis plants (Kwon et al., 2009). Consistent lower abundance of RAB proteins in the LS PCG compared with the ES PCG also highlights the probable role of RAB genes in the senescence processes of this plant.

#### Higher abundance of the calvin cycle and pentose phosphate pathway derived proteins in late senescing PCG

Transketolase, sedoheptulose-1,7-bisphosphatase (SBPase), and fructose-bisphosphate aldolase, all of chloroplastic origin, had increased abundance in LS PCG compared with ES PCG when the fold changes were evaluated before senescence. All three enzymes are part of the Calvin cycle and play a role in important rate limiting steps (Supplementary Figure 4).

As one of the crucial pentose phosphate pathway (PPP) enzymes, transketolase catalyzes the reversible reaction in the formation of ribulose-5-phosphate, and this reaction increases the regeneration rate of RuBisCO (Jones et al., 2012). Transketolase also facilitates isoprenoids synthesis and ultimately reduces oxidative stress (Bouvier et al., 1998, 2005; Lichtenthaler, 1999; Loreto et al., 2001; Vickers et al., 2009). Therefore, the regulation of the PPP and the Calvin cycle seems to be a factor during senescence in plants. Increased abundance of transketolase in LS PCG compared with ES PCG potentially signifies its role in PPP signaling required for cell survival or senescence.

Similarly, SBPase, which produces sedoheptulose-7-phosphate during the Calvin cycle, speeds up the regeneration of RuBisCO with RuBP, thereby speeding up carbon fixation (Lefebvre et al., 2005). An SBPase loss of function mutant in Arabidopsis, *sbp*, showed severe growth retardation, poor SBPase dependent carbon assimilation, and starch biosynthesis (Moore et al., 2003). Moreover, overexpression of *SBPase* in tobacco plants resulted in enhanced photosynthesis and growth rates in the early stage of development (Lefebvre et al., 2005; Feng et al., 2007; Liu et al., 2012), and increased growth rate and accumulation of biomass (Tamoi et al., 2006). Similarly, the higher abundance of SBPase and fructose-bisphosphate aldolase in LS PCG also corroborates the importance of the Calvin cycle during senescence. The overexpression of SBPase, with suitable promoters in tobacco and rice, has been reported to enhance photosynthetic rates and growth rates, whereas the downregulation of this gene resulted in retarding growth (Lefebvre et al., 2005; Tamoi et al., 2006; Feng et al., 2007; Liu et al., 2012). Therefore, the overexpression of the genes responsible for this enzyme may be a good strategy for increasing biomass in biofuel crops, such as PCG and SG. Data from the study of natural senescence of switchgrass flag leaves would support a positive role for SBPase in delaying senescence. SBPase transcript levels decreased significantly in flag leaves with the onset of senescence in field-grown switchgrass plants (Palmer et al., 2015).

Another potential Calvin cycle enzyme, fructose bisphosphate aldolase, regulates photosynthetic carbon flux. Importantly, downregulation of fructose bisphosphate aldolase causes reduced photosynthesis, altered levels of sugar and starch, and retarded growth in potato plants (Haake et al., 1998). Studies on young *Brassica napus* leaves showed that fructose bisphosphate aldolase expression is lower in mature green leaves but is then enhanced again during senescence (Buchanan-Wollaston, 1997). Consistent with earlier reports, our proteomic profiling shows increased abundance of fructose-bisphosphate aldolase in LS PCG, suggesting the significance of Calvin cycle activities in this late senescing genotype. It has also been reported that the overexpression of the *Arabidopsis* plastid fructose bisphosphate aldolase in tobacco plants resulted in augmented biomass by up to 2.2-fold (Uematsu et al., 2012). Producing plants overexpressing the genes encoding this enzyme may be another strategy to produce more biomass in biofuel crops. Biomass accumulation in LS PCG is higher compared with ES PCG (Boe, unpublished). Thus, the accumulation of higher amounts of biomass in LS PCG seems to be the result of upregulation of the Calvin cycle, increased photosynthesis, and increased carbon assimilation.

In C_4_ plants, such as SG and PCG, CO_2_ is generally not the rate-limiting factor as the oxygenase activity of RuBisCO is mostly limited due to high internal CO_2_ concentrations in these plants. As a result, the regeneration rate of RuBisCO plays a critical role in enhancing the photosynthesis rate and increasing biomass. Increased abundance of Calvin cycle enzymes (transketolase, sedoheptulose-1,7-bisphosphatase, and fructose-bisphosphate aldolase) along with increased abundance of photosynthesis machinery (oxygen evolving enhancer proteins, ferrodoxin-NADP-reductase, and the α- and β-subunits of ATP synthase) likely increases the regeneration rate of RuBisCO and the overall carbon assimilation in the LS PCG genotype.

#### Oxidative phosphorylation and senescence

Two key proteins of oxidative phosphorylation, succinate dehydrogenase (Complex II) and cytochrome c oxidase (Complex IV), were found in differential abundance during senescence. Complex IV was found to be more abundant in ES PCG compared with LS PCG before senescence, whereas complex II had increased abundance during senescence in PCG when the protein fold change was analyzed for “after senescence” to “before senescence” (Supplementary Figure 5). Oxidative phosphorylation is well shown to generate ROS in both animal and plant cells (Fleury et al., 2002; Turrens, 2003).

Consistent lower abundance of complex IV of the oxidative phosphorylation cycle (i.e., cytochrome c-oxidase) in LS PCG compared with ES PCG, along with higher abundance of complex II (i.e., succinate dehydrogenase) during senescence in PCG, suggests the probable role played by oxidative phosphorylation pathways in the senescence mechanism of PCG. Upregulation of oxidative phosphorylation may contribute to the generation of ROS, thereby signaling for senescence; thus, its downregulation may play a role in delaying the senescence process, as observed in LS PCG. We also found that during senescence, there was higher abundance of proteins involved in fatty acid break down, proteolysis, starch conversion, and gluconeogenesis. Upregulation of fatty acid breakdown also mediates mitochondrial dysfunction ROS generation, ultimately increasing oxidative stress in plants. Upregulation of genes involved in fatty acid break down, proteolysis, starch conversion, and gluconeogenesis also results in hexose accumulation, which signals for developmental senescence (Dai et al., 1999). Similarly, upregulation of sucrose phosphate synthase during senescence may have played a role in sucrose formation from hexoses and translocation to the sinks, which are rhizomes in the case of these perennial grasses (Lemoine et al., 2013). Many of these changes at the protein level mirror data observed at the transcript level in senescing switchgrass flag leaves (Palmer et al., 2015).

### Prediction of protein-protein interactions

**Figure 7:**
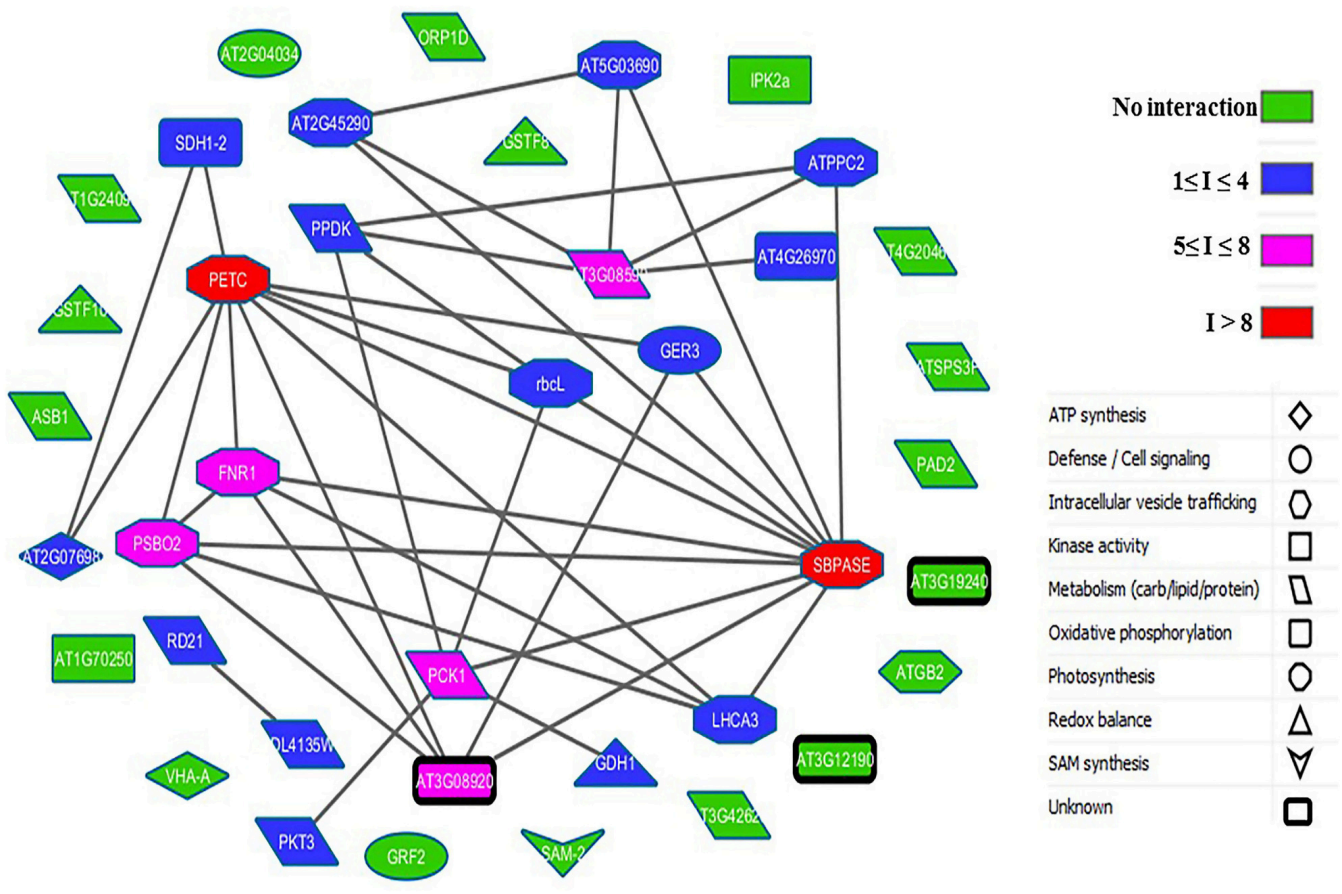
**Protein-protein interaction map for Arabidopsis homologs of differentially abundant proteins found in SG and PCG during senescence**. Number of nodes = 39, number of interactions = 39.

**Figure 8:**
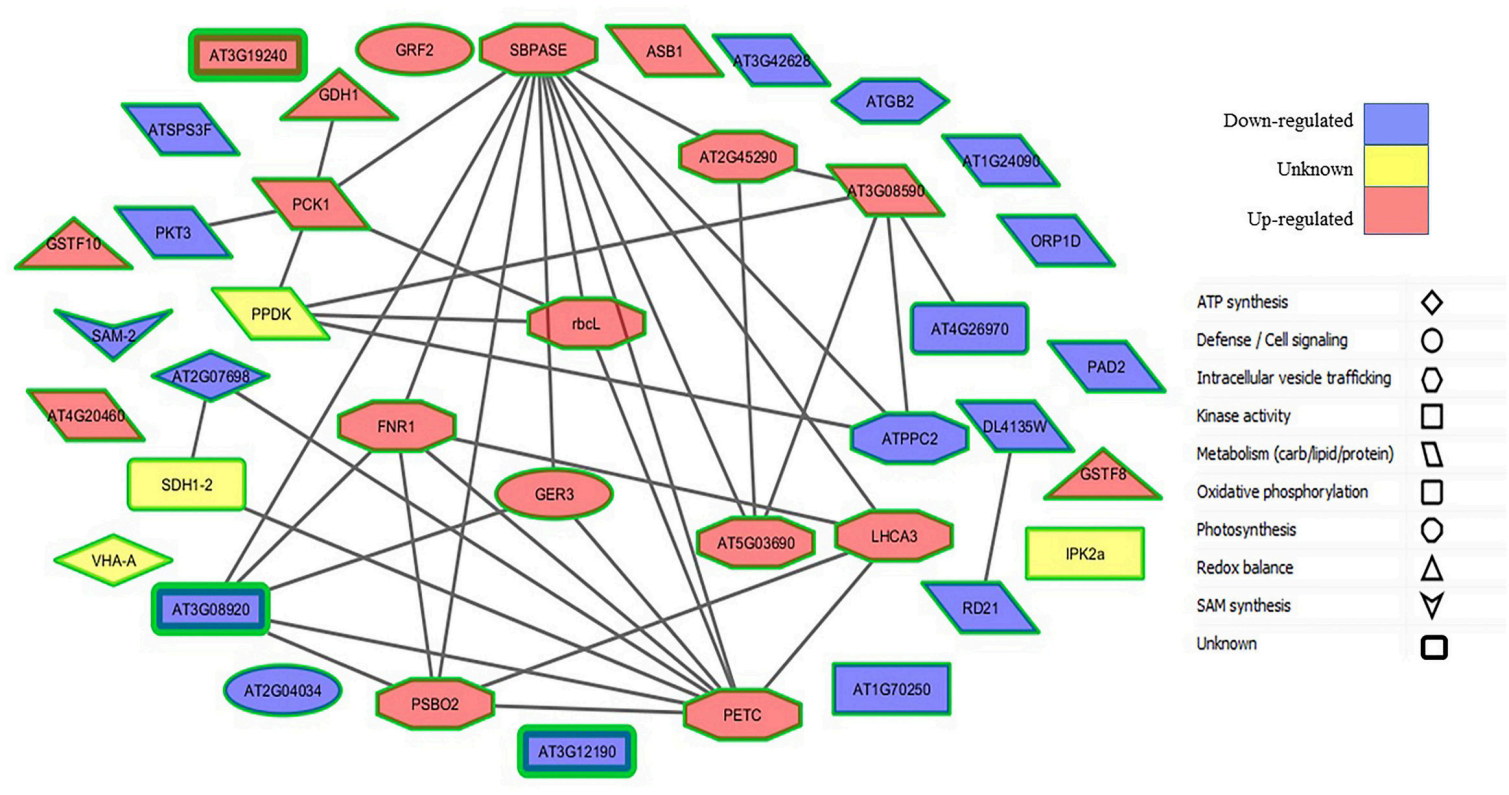
**Protein-protein interaction map for Arabidopsis homologs of differentially abundant proteins observed for LS PCG to ES PCG (G/E) before the onset of senescence**. Red nodes represent proteins which had consistent higher abundance in LS PCG, blue nodes represent proteins with lower abundance, and yellow nodes represent proteins whose fold change in abundance was not significant.

To better understand cellular and molecular functions, it is crucial to address various functional interactions between proteins. Using computational methods (see Section Bioinformatic Analyses), we tried to model a variety of functional protein-protein interactions between the differentially abundant proteins during senescence in PCG and SG to elucidate the complex underlying regulatory processes. From the database search, we found 39 homologous proteins in Arabidopsis (Supplementary Table 3) and analyzed their interactions (Supplementary Table 4). Our computational analysis of protein-protein interaction networks revealed two major proteins (PETC and SBPase) that have more than eight interactions considered to be at the central body of the network system (Figure 7). Most of the proteins central to this network were related to photosynthesis, whereas other connected proteins were involved in metabolism, ATP synthesis, and cell signaling (Supplementary Table 4). The majority of the proteins involved in photosynthesis were more abundant in LS PCG compared with ES PCG before senescence (Figure 8). This suggests that the upregulation of the photosynthesis machinery is a key factor in delaying senescence. Senescence mediated differential expression of genes encoding these central body proteins of the network may ultimately affect the specific pathways of related predicted partner proteins during growth.

## Conclusion

This study identified 69 statistically significant, differentially abundant proteins in PCG and SG genotypes contrasting in senescence, namely ES (early senescence) and LS (late senescence), using 2-Dimensional, differential in-gel electrophoresis (2D-DIGE) followed by mass spectrometry of leaf samples collected just before and after onset of senescence. The goal was to begin a catalog of leaf proteins that could be indicators of either phenotype as a starting point for more proteomic and physiological studies to understand mechanisms impacting senescence in these two biofuel grasses. LS genotypes of both grasses analyzed before senescence contained significantly higher abundances of a 14-3-3 like protein and a glutathione-S-transferase protein when compared to the ES genotypes. The higher abundance of 14-3-3 like proteins may be one factor that impacts the senescence process in both LS PCG and LS SG and provides a target at the protein and genetic level to evaluate in field nurseries containing PCG and SG germplasm. Species specific differences in proteins between the ES and LS genotypes were also observed, indicating that subtle variations in the accumulation of specific proteins could influence longevity. As an example, the maintenance of proteins associated with the photosynthetic machinery in LS PCG compared to ES PCG suggests that factors that control proteolysis within plastids could be important in delaying senescence and boosting biomass yields, especially toward the end of the rowing season. Overall this study opens the door toward a more vibrant understanding of various differentially abundant senescence-related proteins and should prove valuable to the future improvement of perennial grasses for higher biomass yields (Kim et al., 2014; Das et al., 2015). Application of such gel-based quantitative proteomic approaches together with mapping of post-translational modifications will provide comprehensive insights into the regulation of various senescence-related proteins, corresponding to their biological function (Komatsu et al., 2013).

## Author contributions

AB, GS, JG, PR, and JR designed the experiments. BP, AD, MT performed the experiments. BP, AD, GS, JG, PR, NP, and JR analyzed the results. BP, AD, AB, NP, GS, JG, and JR wrote the manuscript.

## Funding

This research was supported by funding from the North Central Regional Sun Grant Center at South Dakota State University through a grant provided by the US Department of Agriculture under award number 2010-38502-21861. This work was supported in part by the USDA-ARS CRIS project 3042-21000-030-00D and CRIS project SD00H541-15. The U.S. Department of Agriculture, Agricultural Research Service, is an equal opportunity/affirmative action employer and all agency services are available without discrimination. Mention of commercial products and organizations in this manuscript is solely to provide specific information. It does not constitute endorsement by USDA-ARS over other products and organizations not mentioned.

### Conflict of interest statement

The authors declare that the research was conducted in the absence of any commercial or financial relationships that could be construed as a potential conflict of interest.

## Supplementary material

Supplementary Figure 1 **Pre- and post-senescence periods were determined by measuring the chlorophyll content**. Red arrow indicates the mid line between pre- and post-senescence period that was determined on the basis of chlorophyll measurement. Sample interval indicates number of weeks and CCI indicates relative amount of chlorophyll content in that particular sample.

Supplementary Figure 2 **Proposed model of sugar metabolism toward hexoses and sucrose formation during senescence which signals for early floral development, translocation of sugars from source to sink, and early senescence**. Analysis revealed higher abundance of five proteins shown above during senescence, whereas three proteins (β-ketoadepyl CoA thiolase, cysteine protease, and sucrose phosphate synthase) had consistent lower abundance in LS PCG compared to ES PCG when analyzed before senescence onset.

Supplementary Figure 3 **Three proteins (oxygen evolving enhancer protein of photosystem II, ferrodoxin-NADP-reductase, and **α**- and **β**-subunit of ATP synthase) found with consistently higher abundance in LS PCG compared to ES PCG that are involved in key steps of photosynthesis**. Figure of photosynthesis reproduced from KEGG reference pathway (http://www.genome.jp/kegg/pathway/map/map00195.html).

Supplementary Figure 4 **Proteins of the Calvin cycle found in greater abundance in LS PCG compared to ES PCG that catalyze the vital rate limiting steps are marked with red arrows**. These enzymes are 1- fructose bishphsphate aldolase, 2- transketolase, 3- sedoheptulose-1,7-bisphosphatase.

Supplementary Figure 5 **Increased abundance of complex II, and complex IV of the oxidative phosphorylation pathway as observed in our study**. Higher abundance of these complexes may contribute to increased ROS levels, thus signaling the expression of senescence associated genes. Consistent higher abundance of complex IV was observed in ES PCG compared LS PCG. Figure of oxidative phosphorylation reproduced from KEGG pathway (http://www.genome.jp/kegg/pathway/map/map00190.html).

Supplementary Table 1 **Fold change in differential abundance of protein spots for after to before senescence, in Early and Late PCG**. F/E represents ratio for after to before senescence in Early PCG, whereas H/G represents that ratio for late PCG. Positive values represent increased abundance; negative values represent decreased abundance. All ratios included are statistically significant (*p* < 0.01).

Supplementary Table 2 **Fold change in differential abundance of protein spots for after to before senescence, in Early and Late SG**. B/A represents ratio for after to before senescence in Early SG, whereas D/C represents that ratio for late SG. Positive values represent increased abundance; negative values represent decreased abundance. All ratios included are statistically significant (*p* < 0.01). Match quality H means high confidence, L means low confidence and N means no confidence. ^*^NA, not available.

Supplementary Table 3 **Proteins, and their Arabidopsis homologs and percentage similarity**.

Supplementary Table 4 **Protein function in protein-protein interaction table**.

Supplementary Table 5 **Protein identifications with NCBInr database search**.

Supplementary Table 6 **Protein identifications with Phytozome v10.3 database search**.
