# Supplementary material for: Proteomic Responses of Switchgrass and Prairie Cordgrass to Senescence
Source: Front Plant Sci. 2016 Mar 14;7:293. doi: 10.3389/fpls.2016.00293 (PMC4789367; doi:10.3389/fpls.2016.00293)
Supplement: Supplementary Figure 1 — Pre- and post-senescence periods were determined by measuring the chlorophyll content. Red arrow indicates the mid line between pre- and post-senescence period that was determined on the basis of chlorophyll measurement. Sample interval indicates number of weeks and CCI indicates relative amount of chlorophyll content in that particular sample. [file Presentation1.PPTX]

## Slide 1
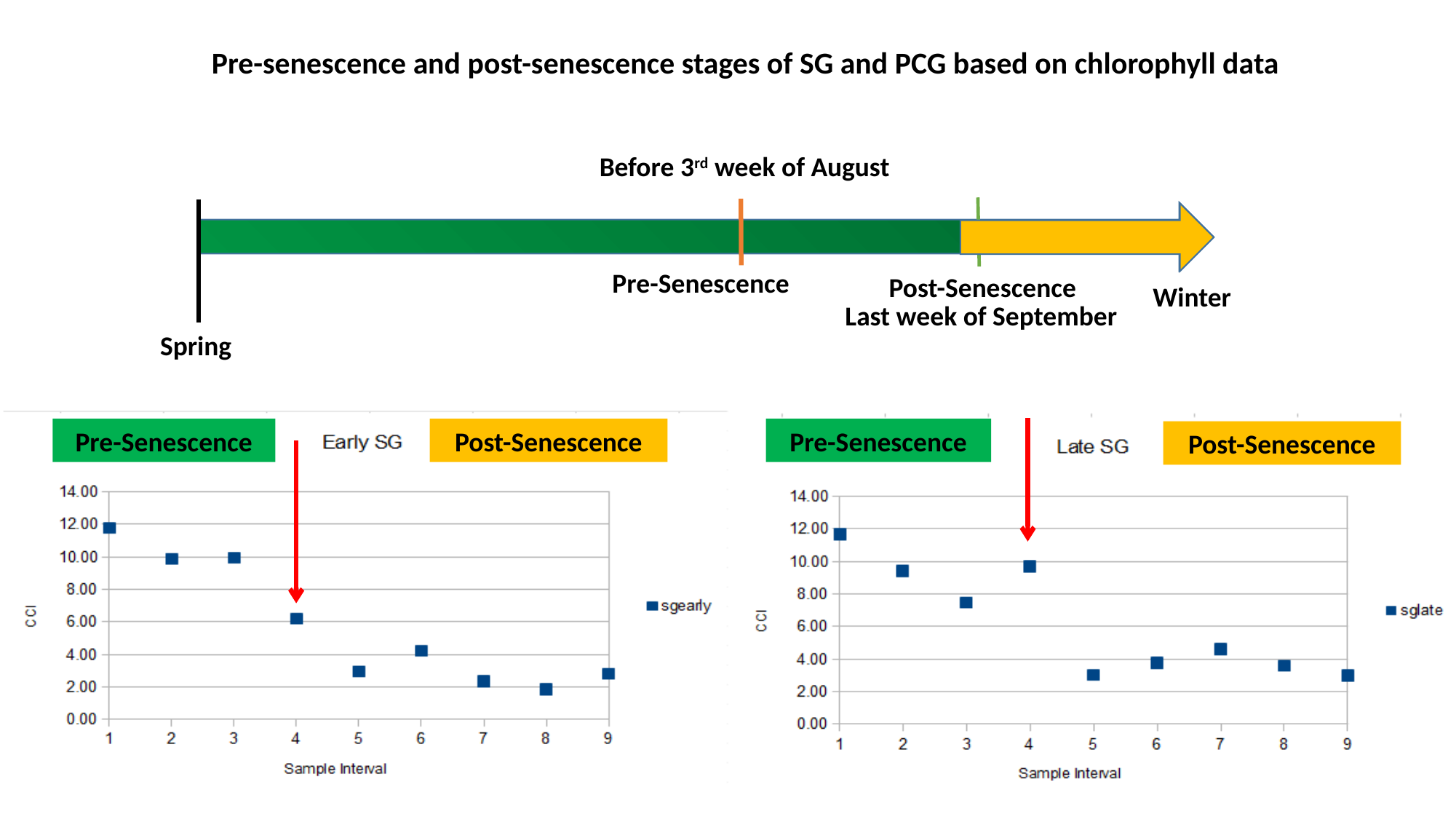

Pre-senescence and post-senescence stages of SG and PCG based on chlorophyll data
Before 3rd week of August
Pre-Senescence
Post-Senescence
Winter
Last week of September
Spring
Pre-Senescence
Post-Senescence
Pre-Senescence
Post-Senescence

## Slide 2
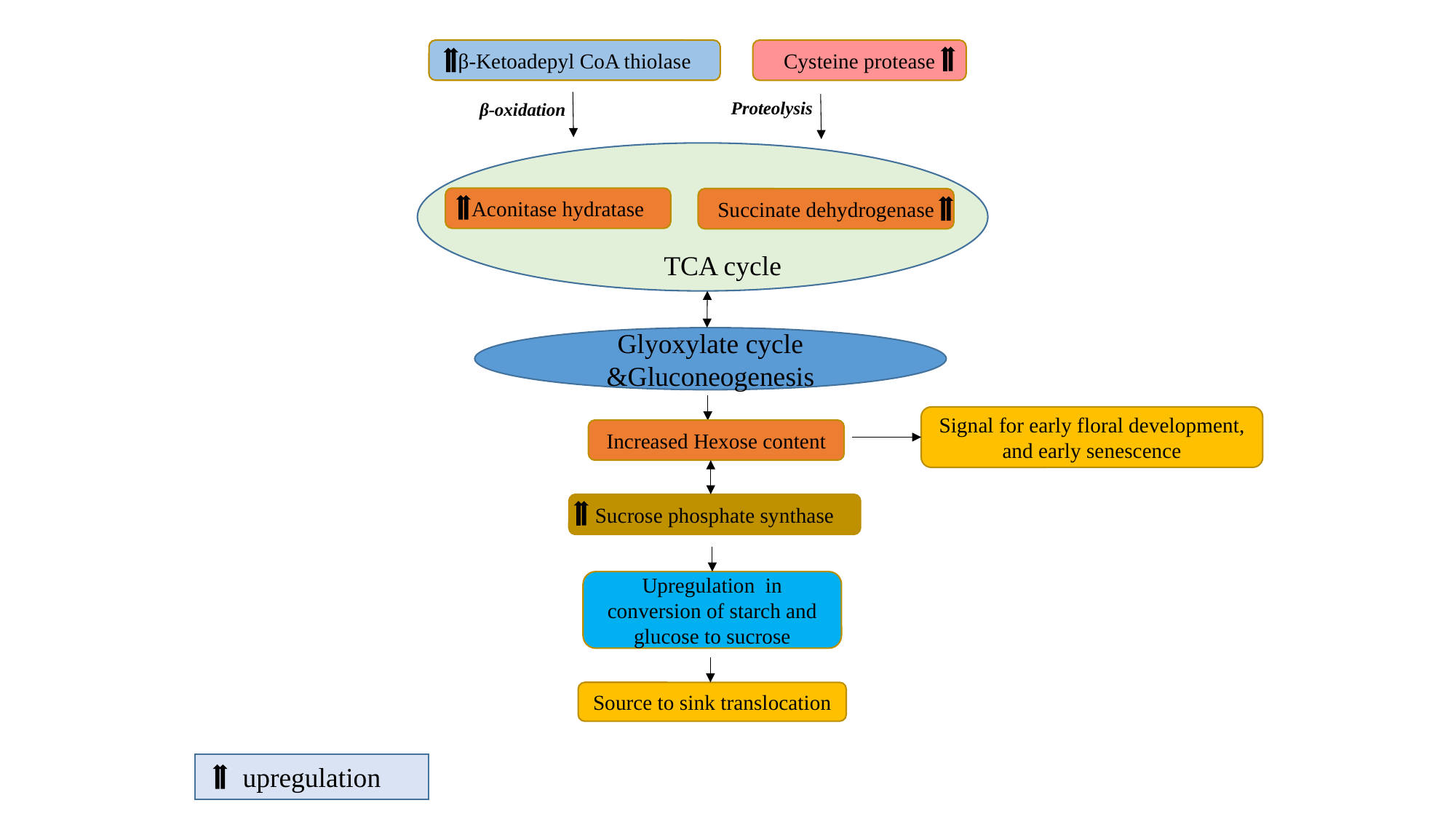

β-Ketoadepyl CoA thiolase
Cysteine protease
Proteolysis
β-oxidation
Aconitase hydratase
Succinate dehydrogenase
TCA cycle
Glyoxylate cycle &Gluconeogenesis
Signal for early floral development, and early senescence
Increased Hexose content
Sucrose phosphate synthase
Upregulation in conversion of starch and glucose to sucrose
Source to sink translocation
upregulation

## Slide 3
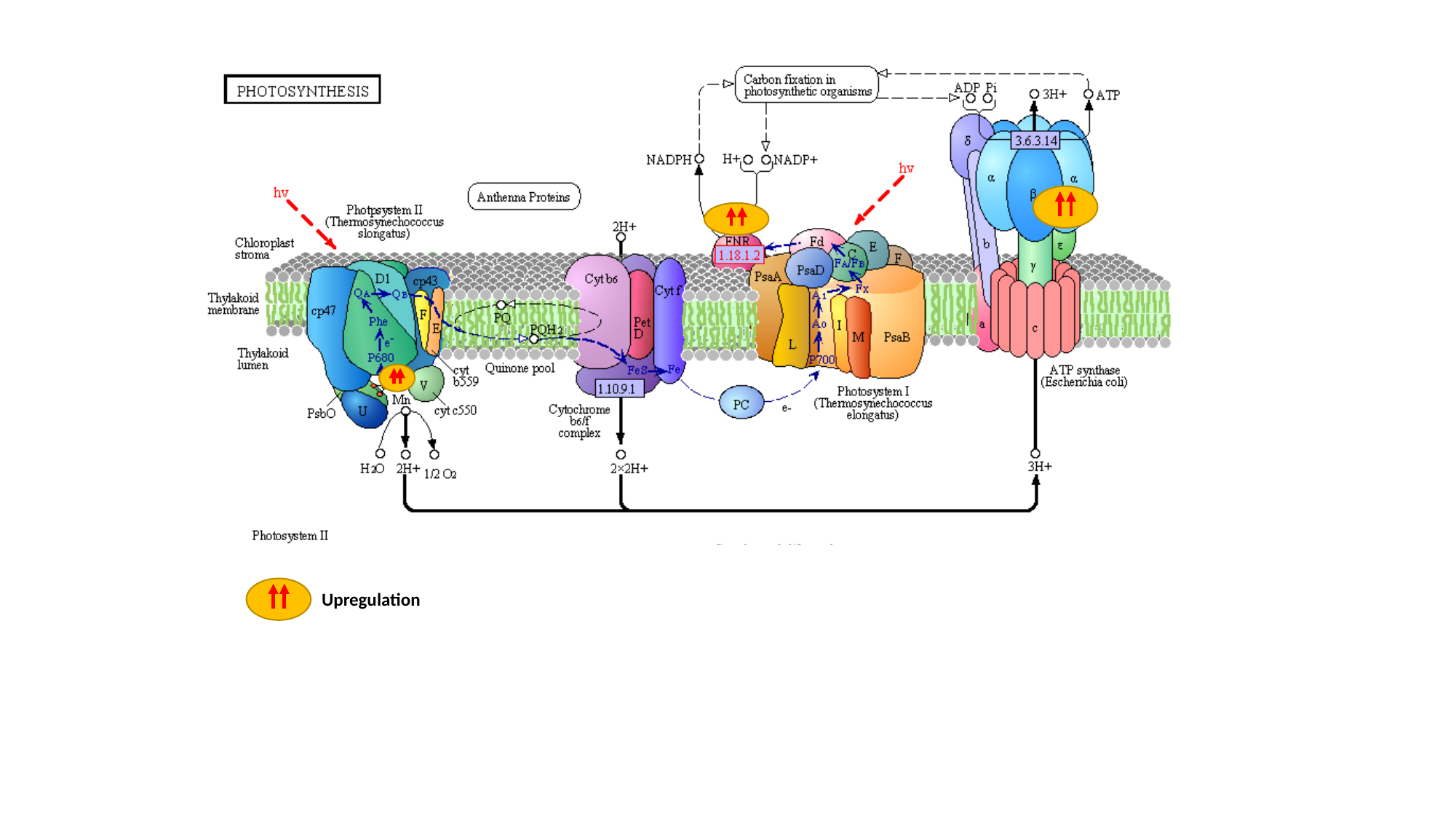

Upregulation

## Slide 4
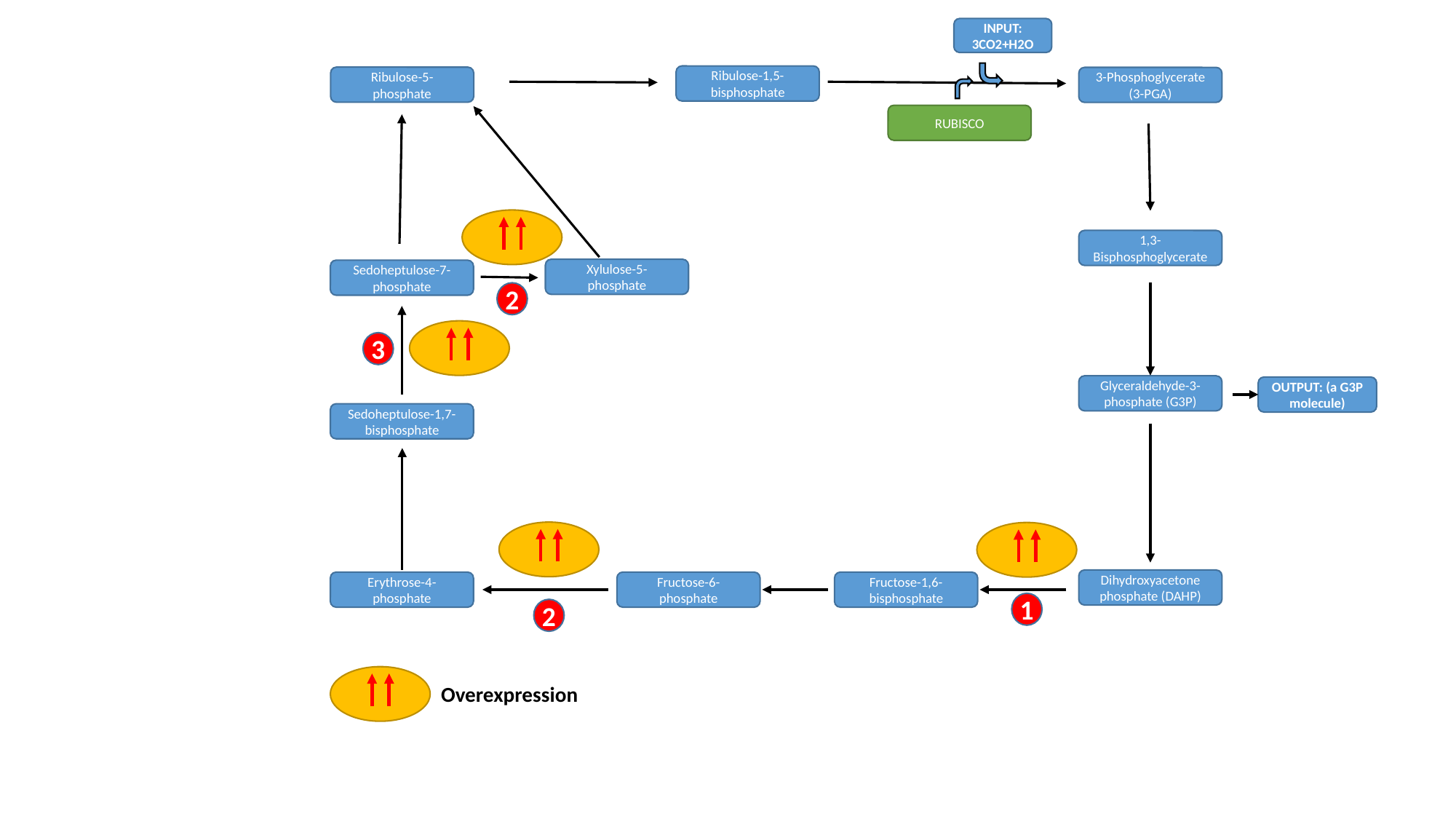

INPUT: 3CO2+H2O
Ribulose-1,5-bisphosphate
Ribulose-5-phosphate
3-Phosphoglycerate (3-PGA)
RUBISCO
1,3-Bisphosphoglycerate
Xylulose-5-phosphate
Sedoheptulose-7-phosphate
2
3
Glyceraldehyde-3-phosphate (G3P)
OUTPUT: (a G3P molecule)
Sedoheptulose-1,7-bisphosphate
Dihydroxyacetone phosphate (DAHP)
Erythrose-4-phosphate
Fructose-6-phosphate
Fructose-1,6-bisphosphate
1
2
Overexpression

## Slide 5
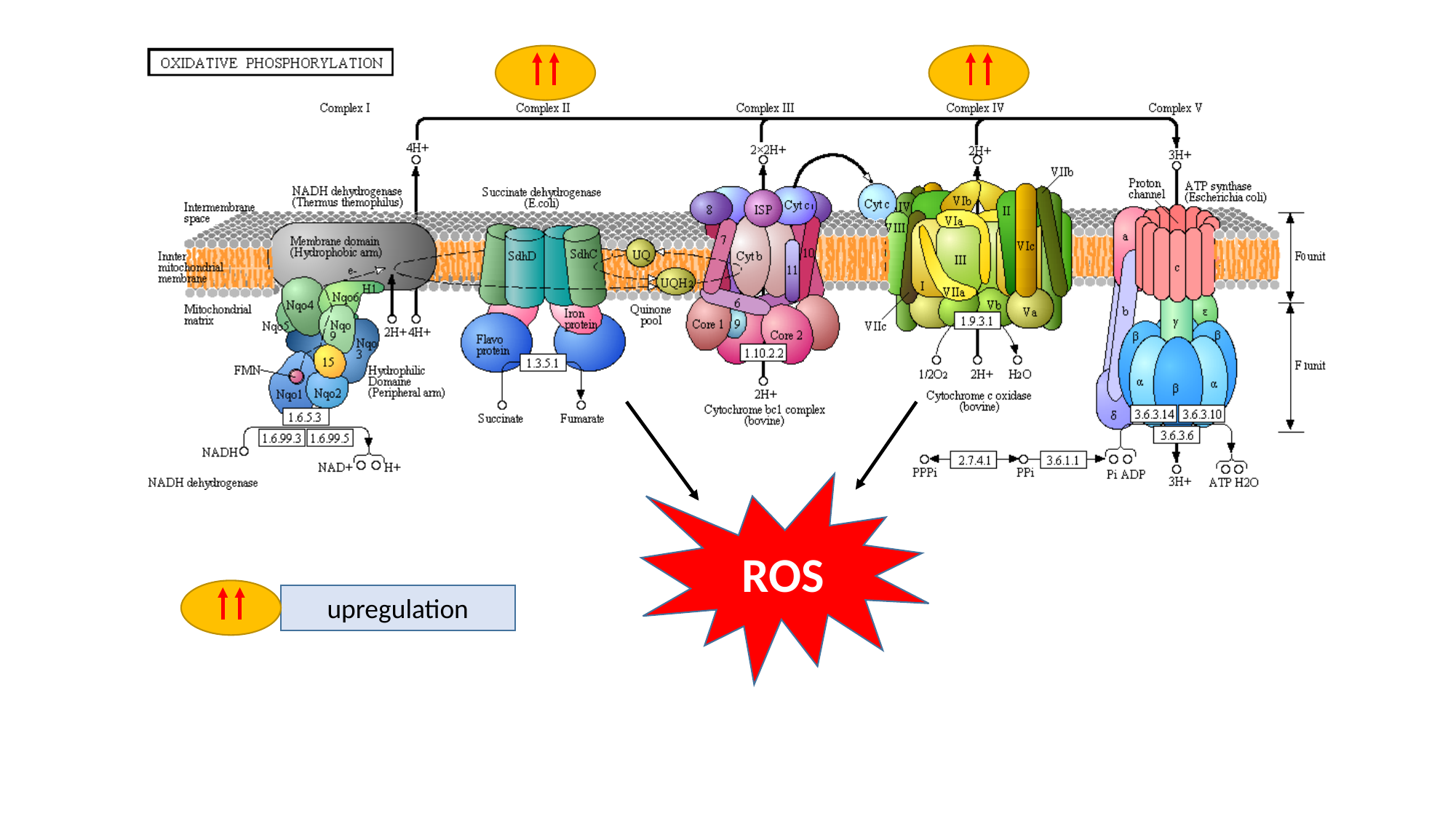

ROS
upregulation
