# Supplementary material for: Proteomic Responses of Switchgrass and Prairie Cordgrass to Senescence
Source: Front Plant Sci. 2016 Mar 14;7:293. doi: 10.3389/fpls.2016.00293 (PMC4789367; doi:10.3389/fpls.2016.00293)
Supplement: Supplementary Table 1 — Fold change in differential abundance of protein spots for after to before senescence, in Early and Late PCG. F/E represents ratio for after to before senescence in Early PCG, whereas H/G represents that ratio for late PCG. Positive values represent increased abundance; negative values represent decreased abundance. All ratios included are statistically significant (p < 0.01). [file Table1.DOCX]

**Supplementary Table 1:** Fold change in differential abundance of protein spots for after to before senescence, in Early and Late PCG. F/E represents ratio for after to before senescence in Early PCG, whereas H/G represents that ratio for late PCG. Positive values represent increased abundance; negative values represent decreased abundance. All ratios included are statistically significant (p<0.01).

| Spot number | **Best hit protein** | **Accession No.** | **F/E Av. Ratio** | **H/G Av. Ratio** | **Protein MW** | **Pep.Count** | **Protein Score** | **Protein Score C.I.%** | **Total Ion Score** | **Total Ion C.I.%** |
| --- | --- | --- | --- | --- | --- | --- | --- | --- | --- | --- |
| 25 | 2,3-bisphosphoglycerate-independent phosphoglycerate mutase | PMGI_MAIZE | 1.87 | 2.3 | 60,582 | 10 | 111 | 100 | 76 | 100 |
| 31 | Succinate dehydrogenase [ubiquinone] flavoprotein subunit, mitochondrial | DHSA_ORYSJ | 2.02 | 1.54 | 68,810 | 10 | 57 | 93 | 29 | 82 |
| 33 | phosphoenolpyruvate carboxykinase [Spartina anglica] | gi\|220938712 | -2.4 | -4.72 | 55,617 | 19 | 543 | 100 | 428 | 100 |
| 34 | phosphoenolpyruvate carboxykinase [Spartina anglica] | gi\|220938712 | -2.56 | -4.88 | 55,617 | 18 | 416 | 100 | 306 | 100 |
| 36 | phosphoenolpyruvate carboxykinase [Spartina anglica] | gi\|220938712 | -1.39 | -5.75 | 55,617 | 23 | 740 | 100 | 572 | 100 |
| 58 | Ribulose bisphosphate carboxylase large chain | RBL_SETIT | -1.42 | -3.08 | 52,648 | 23 | 672 | 100 | 504 | 100 |
| 59 | Ribulose bisphosphate carboxylase large chain | RBL_SETIT | -1.54 | -4.11 | 52,648 | 23 | 715 | 100 | 547 | 100 |
| 60 | Ribulose bisphosphate carboxylase large chain | RBL_AVESA | -1.47 | -5.04 | 52,901 | 22 | 706 | 100 | 544 | 100 |
| 97 | cysteine protease 1 precursor [Zea mays] | gi\|226496089 | 1.83 | 5.06 | 50,730 | 3 | 185 | 100 | 178 | 100 |
| 120 | cysteine protease 1 precursor [Zea mays] | gi\|226496089 | 1.49 | 4.45 | 50,730 | 3 | 155 | 100 | 149 | 100 |
| 128 | unknown [Zea mays] | gi\|223974857 | 1.55 | 1.46 | 39,043 | 7 | 101 | 100 | 70 | 100 |
| 133 | Oxygen-evolving enhancer protein 1, chloroplastic | PSBO_HELAN | -1.46 | -1.54 | 34,202 | 9 | 446 | 100 | 397 | 100 |
| 140 | glutathione S-transferase GSTF14 [Oryza sativa Japonica Group] | gi\|46276327 | 1.94 | 1.27 | 30,710 | 5 | 125 | 100 | 107 | 100 |
| 208 | hypothetical protein azo3784 [Azoarcus sp. BH72] | gi\|119900073 | -10.59 | -1.79 | 12,288 | 2 | 70 | 18 | 61 | 97 |
| 284 | Ribulose bisphosphate carboxylase large chain | RBL_LIQST | -1.62 | -4.26 | 52,626 | 27 | 690 | 100 | 458 | 100 |
