# Supplementary material for: Proteomic Responses of Switchgrass and Prairie Cordgrass to Senescence
Source: Front Plant Sci. 2016 Mar 14;7:293. doi: 10.3389/fpls.2016.00293 (PMC4789367; doi:10.3389/fpls.2016.00293)
Supplement: Supplementary Table 2 — Fold change in differential abundance of protein spots for after to before senescence, in Early and Late SG. B/A represents ratio for after to before senescence in Early SG, whereas D/C represents that ratio for late SG. Positive values represent increased abundance; negative values represent decreased abundance. All ratios included are statistically significant (p < 0.01). Match quality H means high confidence, L means low confidence and N means no confidence. *NA, not available. [file Table2.DOCX]

**Supplementary Table 2**: Fold change in differential abundance of protein spots for after to before senescence, in Early and Late SG. B/A represents ratio for after to before senescence in Early SG, whereas D/C represents that ratio for late SG. Positive values represent increased abundance; negative values represent decreased abundance. All ratios included are statistically significant (p<0.01). Match quality H means high confidence, L means low confidence and N means no confidence. *NA: not available.

| **Spot number** | **MALDI well number** | **Match Quality** | **Best hit protein** | **Accession No.** | **B/A Av. Ratio** | **D/C Av. Ratio** | **Protein MW** | **Protein PI** | **Pep.Count** | **Protein Score** | **Protein Score C.I.%** | **Total Ion Score** | **Total Ion C.I.%** |
| --- | --- | --- | --- | --- | --- | --- | --- | --- | --- | --- | --- | --- | --- |
| **2** | **E2** | L | Putative aconitate hydratase, cytoplasmic OS=Oryza sativa subsp. japonica GN=Os08g0191100 PE=3 SV=1 | ACOC_ORYSJ | 2.04 | 1.53 | 98,021 | 5.7 | 13 | 56 | 92 | 19 | 0 |
| **15** | **E10** | H | Transketolase, chloroplastic OS=Zea mays PE=1 SV=1 | TKTC_MAIZE | -4.04 | -1.73 | 72,948 | 5.5 | 7 | 102 | 100 | 85 | 100 |
| **16** | **E11** | H | Transketolase, chloroplastic OS=Zea mays PE=1 SV=1 | TKTC_MAIZE | -4.3 | -1.84 | 72,948 | 5.5 | 7 | 92 | 100 | 75 | 100 |
| **19** | **E12** | H | Transketolase, chloroplastic OS=Zea mays PE=1 SV=1 | TKTC_MAIZE | -1.29 | -1.15 | 72,948 | 5.5 | 6 | 72 | 100 | 60 | 100 |
| **45** | **E24** | H | ATP synthase subunit alpha, chloroplastic OS=Saccharum officinarum GN=atpA PE=3 SV=1 | ATPA_SACOF | -2.51 | -1.72 | 55,656 | 5.9 | 26 | 735 | 100 | 516 | 100 |
| **46** | **F1** | H | ATP synthase subunit alpha, chloroplastic OS=Saccharum hybrid GN=atpA PE=2 SV=2 | ATPA_SACHY | -3.58 | -1.78 | 55,716 | 5.9 | 31 | 981 | 100 | 694 | 100 |
| **48** | **F2** | H | ATP synthase subunit alpha, chloroplastic OS=Saccharum officinarum GN=atpA PE=3 SV=1 | ATPA_SACOF | -4.09 | -2.12 | 55,656 | 5.9 | 30 | 874 | 100 | 602 | 100 |
| **56** | **F6** | H | Ribulose bisphosphate carboxylase large chain (Fragment) OS=Securidaca diversifolia GN=rbcL PE=3 SV | RBL_SECDI | -1.92 | -2.62 | 51,600 | 6.2 | 11 | 96 | 100 | 46 | 100 |
| **58** | **F7** | H | Ribulose bisphosphate carboxylase large chain OS=Setaria italica GN=rbcL PE=3 SV=1 | RBL_SETIT | -9.61 | -3.13 | 52,648 | 6.4 | 23 | 672 | 100 | 504 | 100 |
| **59** | **F8** | H | Ribulose bisphosphate carboxylase large chain OS=Setaria italica GN=rbcL PE=3 SV=1 | RBL_SETIT | -14.72 | -4.4 | 52,648 | 6.4 | 23 | 715 | 100 | 547 | 100 |
| **60** | **F9** | H | Ribulose bisphosphate carboxylase large chain OS=Avena sativa GN=rbcL PE=3 SV=1 | RBL_AVESA | -4.13 | -1.73 | 52,901 | 5.9 | 22 | 706 | 100 | 544 | 100 |
| **98** | **F14** | H | Sedoheptulose-1,7-bisphosphatase, chloroplastic OS=Triticum aestivum PE=2 SV=1 | S17P_WHEAT | -4.23 | -1.91 | 42,034 | 6.0 | 7 | 304 | 100 | 275 | 100 |
| **115** | **F15** | H | Fructose-bisphosphate aldolase, chloroplastic OS=Oryza sativa subsp. japonica GN=Os11g0171300 PE=1 | ALFC_ORYSJ | -4.6 | -2.12 | 41,980 | 6.4 | 11 | 194 | 100 | 136 | 100 |
| **127** | **F17** | N | Ribulose bisphosphate carboxylase large chain (Fragment) OS=Securidaca diversifolia GN=rbcL PE=3 SV | RBL_SECDI | -1.04 | -1.11 | 51,600 | 6.2 | 9 | 46 | 21 | 12 | 0 |
| **128** | **F18** | H | unknown [Zea mays] | gi\|223974857 | 17.36 | 5.95 | 39,043 | 7.9 | 7 | 101 | 100 | 70 | 100 |
| **133** | **F20** | H | Oxygen-evolving enhancer protein 1, chloroplastic OS=Helianthus annuus GN=PSBO PE=1 SV=1 | PSBO_HELAN | -4.61 | -2.1 | 34,202 | 5.4 | 9 | 446 | 100 | 397 | 100 |
| **134** | **F21** | H | Oxygen-evolving enhancer protein 1, chloroplastic OS=Solanum lycopersicum GN=PSBO PE=2 SV=2 | PSBO_SOLLC | -3.54 | -1.79 | 34,926 | 5.9 | 6 | 171 | 100 | 147 | 100 |
| **140** | **F22** | H | glutathione S-transferase GSTF14 [Oryza sativa Japonica Group] | gi\|46276327 | 5.17 | 2.64 | 30,710 | 0 | 5 | 125 | 100 | 107 | 100 |
| **142** | **F23** | N | NAD-dependent epimerase/dehydratase [Zea mays] | gi\|226499246 | 6.02 | 2.32 | 27,752 | 0 | 9 | 60 | 0 | 11 | 0 |
| **225** | **G7** | N | Oxysterol-binding protein-related protein 1D OS=Arabidopsis thaliana GN=ORP1D PE=2 SV=1 | ORP1D_ARATH | 1.2 | 1.3 | 92,274 | 6.1 | 13 | 40 | 0 | NA | NA |
| **230** | **G9** | N | S-adenosylmethionine synthase 1 OS=Brassica juncea GN=SAMS1 PE=2 SV=1 | METK1_BRAJU | 1.05 | 1.23 | 43,199 | 5.5 | 9 | 42 | 0 | NA | NA |
| **237** | **G10** | N | Protein CYPRO4 OS=Cynara cardunculus GN=CYPRO4 PE=2 SV=1 | CYPR4_CYNCA | -2.07 | -1.33 | 55,611 | 7.6 | 11 | 44 | 0 | NA | NA |
| **284** | **G16** | H | Ribulose bisphosphate carboxylase large chain OS=Liquidambar styraciflua GN=rbcL PE=3 SV=1 | RBL_LIQST | -9.77 | -2.88 | 52,626 | 6.0 | 27 | 690 | 100 | 458 | 100 |
| **287** | **G18** | H | predicted protein [Hordeum vulgare subsp. vulgare] | gi\|326529055 | 1.71 | 1.4 | 44,226 | 0 | 11 | 82 | 99 | NA | NA |
| **291** | **G19** | H | cysteine proteinase Mir3 precursor [Zea mays] | gi\|162463464 | 3.31 | 1.58 | 51,754 | 5.8 | 4 | 90 | 99 | 82 | 100 |
| **314** | **G24** | H | chloroplast PSI type III chlorophyll a/b-binding protein [Helianthus annuus] | gi\|159138869 | -4.12 | -1.82 | 13,851 | 0 | 6 | 297 | 100 | 256 | 100 |
| **316** | **H1** | L | hypothetical protein VITISV_010874 [Vitis vinifera] | gi\|147843505 | 3.59 | 1.78 | 37,033 | 0 | 11 | 70 | 89 | NA | NA |
| **317** | **H2** | H | PREDICTED: glutathione S-transferase F8, chloroplastic-like [Brachypodium distachyon] | gi\|357114170 | 2.59 | 1.2 | 25,819 | 0 | 7 | 136 | 100 | 100 | 100 |
