# Supplementary material for: Proteomic Responses of Switchgrass and Prairie Cordgrass to Senescence
Source: Front Plant Sci. 2016 Mar 14;7:293. doi: 10.3389/fpls.2016.00293 (PMC4789367; doi:10.3389/fpls.2016.00293)
Supplement: Supplementary Table 3 — Proteins, and their Arabidopsis homologs and percentage similarity. [file Table3.DOCX]

**Supplementary Table 3**: Proteins, and their Arabidopsis homologs and percentage similarity
